# Metal Matrix Composite Coatings with Polymer Fillers: Fabrication Technologies, Properties, and Applications

**DOI:** 10.3390/polym18212611

**Published:** 2026-10-22

**Authors:** Natalia A. Shapagina, Vladimir V. Dushik

**Affiliations:** Frumkin Institute of Physical Chemistry and Electrochemistry, Russian Academy of Sciences, Leninsky Prospect 31-4, 119071 Moscow, Russia; v.dushik@gmail.com

**Keywords:** MMPC coatings, metal matrix, polymer filler, electrodeposition, electroless plating, thermal spraying, cold spray, hybrid technologies, interfacial adhesion, MMPC properties

## Abstract

Metal matrix composite coatings with polymer fillers are a rapidly developing class of multifunctional materials that combine the mechanical strength, electrical conductivity, and corrosion resistance of metals with the low coefficient of friction, chemical inertness, and functional versatility of polymers. This review provides a systematic analysis of metal matrix polymer composite (MMPC) coatings, covering their classification according to the metallic matrix material (Ni, Cu, Co, Al, Ti, and Zn), the type of polymer filler (fluoropolymers, thermosetting resins, biopolymers, and electrically conductive polymers), and filler morphology (particles, fibers, and microcapsules). The main fabrication technologies are considered in detail, including electrodeposition, electroless deposition, thermal spraying, and cold spray, as well as hybrid and additive methods, with a comparative analysis of their advantages, limitations, and fields of application. The effects of filler composition, particle size and concentration, and the nature of the metal–polymer interface on the structure and functional properties of the coatings are discussed, including tribological characteristics, corrosion resistance, mechanical properties, and specific functionalities such as superhydrophobicity, anti-icing, biocompatibility, and self-healing. Unlike most existing reviews, which focus either on a single fabrication method, typically electrodeposition, or on a specific metal/polymer pair, this work provides a comprehensive cross-comparison of all key technologies, including hybrid approaches and additive manufacturing. The review also systematizes unresolved challenges, including weak adhesion at the metal/polymer interface, limitations in thermal resistance, difficulties in scaling up production, and the lack of standardized testing methods, while outlining promising directions such as nanostructured fillers, self-healing systems, and environmentally friendly manufacturing technologies. This review will be useful for materials scientists, process engineers, and surface engineering specialists working in mechanical engineering, aerospace, the oil and gas sector, medical device manufacturing, and electronics, as well as for researchers developing next-generation functional coatings. The work can serve as a guide for selecting optimal matrix/filler combinations and deposition methods for specific service conditions and as a starting point for designing MMPCs with a predefined combination of multifunctional properties.

## 1. Introduction

A composite material (CM) is an artificially created heterogeneous continuous system consisting of two or more components that retain their individual properties and are separated by distinct interfaces. Their combined use makes it possible to obtain a combination of properties that cannot be achieved when each component is used separately [1,2]. A composite consists of a matrix (the base that provides structural integrity and load transfer) and a filler (the reinforcing phase responsible for specific properties) [1]. CMs are classified according to the nature of the matrix (polymer, metallic, or ceramic) and their structure (fiber-reinforced (1D structures), particulate-filled (0D structures), and layered (2D structures)). This typology reflects the nominal dimensionality of the structural elements; however, the structure of real CMs is often hybrid. For example, layers may contain dispersed particles or short fibers, and therefore a material cannot always be strictly assigned to one of the categories listed above [3,4,5,6].

Today, CMs are used in a wide variety of forms, such as bulk materials, coatings, and others, and in numerous fields, including structural components, mechanical engineering, construction, aviation, medicine, sports, and other industries. The choice of CM form depends on the specific requirements for the product, service conditions, and available manufacturing technologies. In the context of this review, the focus is on metal matrix composite coatings with polymer fillers, which combine the advantages of metals (strength, stiffness, and electrical conductivity) and polymers (flexibility, resistance to aggressive environments, and reduced structural weight). Such coatings are used in mechanical engineering, aerospace, electronics, medicine, and other fields [7,8,9].

A metal matrix composite coating with a polymer filler is a coating in which a continuous metallic phase (matrix) serves as the base, while dispersed polymer particles (filler) are incorporated into it to impart new functional characteristics. Such coatings belong to the class of particulate-filled metal matrix composites, in which a polymer dispersed phase is uniformly distributed throughout the metallic matrix. In these coatings, the matrix is most commonly composed of Ni, Cu, Zn, Co, Al, and their alloys, and less frequently Ti, Mg, or steel. Various thermoplastics and thermosetting polymers are used as polymer fillers, with their selection depending on the required properties of the composite (strength, elasticity, thermal resistance, chemical resistance, etc.). These include epoxy and phenol-formaldehyde resins, polyamides, polytetrafluoroethylene, polyvinyl chloride, polyurethanes, polyimides, polystyrene, polypropylene, polyethylene, and other thermoplastics, as well as other polymers. In addition, the review covers related architectures in which a continuous metallic phase contains a polymer component in a non-dispersed form—polymer film interlayers (e.g., UAM laminates) and polymer-impregnated porous metallic coatings. These are considered extended MMPC architectures and are explicitly distinguished from “polymer-on-metal” systems, where a polymer layer is applied onto a metallic substrate without continuous metal encapsulation.

Metal matrix composite coatings with polymer fillers represent a promising direction in the development of advanced materials. Their relevance is associated with the possibility of combining the advantages of metals (high strength, stiffness, and electrical conductivity) and polymers (low coefficient of friction, chemical resistance, and reduced structural weight) [9,10,11,12,13,14]. With the rapid development of mechanical engineering, the aerospace industry, and medicine, the demand for such materials is constantly increasing, while increasing attention is being paid to environmental aspects of materials science. In particular, the use of recycled polymer components and polymers obtained through plastic waste processing is considered promising, as it makes it possible to combine the functional advantages of composites with addressing the problem of plastic waste disposal. At the same time, the direct use of microplastics from natural waters (including the Pacific Ocean) as a filler is not considered justified: due to contamination, heterogeneity, and toxicological risks, such material is unsuitable for controlled coating production [15,16,17].

Traditional coatings based on pure metals may have limitations in terms of their overall performance under modern service conditions. Limitations in wear resistance, corrosion resistance, and tribological properties necessitate the development of new approaches to the fabrication of protective coatings. The polymer component in a composite coating plays a key role in determining the functional properties of the material. It provides reduced friction, improved wear resistance, corrosion protection, thermal conductivity control, and the possibility of imparting specific properties such as hydrophobicity and biocompatibility [1,9,18,19,20,21].

The main objective of this review is to systematize current knowledge of metal matrix polymer composite (MMPC) materials and their coatings, analyze their fabrication methods, and examine their properties. To achieve this objective, the following tasks must be addressed: to classify the existing types of MMPCs (Section 2), analyze methods for fabricating MMPCs and their coatings (Section 3), investigate the effect of composition on their properties (Section 4, Section 5 and Section 6), identify areas of effective application (Section 7), determine promising directions for further development (Section 8 and Section 9) and to present, in a dedicated Section 10, a comprehensive analysis and synthesis of the key findings from the reviewed research, highlighting the main trends, achievements, and gaps in the field of MMPC coatings.

The literature for this review was selected through a systematic search across the Scopus, Web of Science, ScienceDirect, and Google Scholar databases, covering publications from 2000 to 2025. The search was conducted using keywords and their combinations related to metal–polymer composites, metallic matrices, polymer phases, coating deposition methods, and specific metal–polymer systems, with a gradual narrowing of the search scope. Additionally, manual selection of relevant publications and analysis of their bibliographic lists were employed. Experimental, theoretical, and review papers in English and Russian were considered, provided they were devoted to composite coatings with a continuous metallic matrix and a dispersed polymer phase and described the composition, structure, properties, or fabrication methods of such coatings. Particular attention was paid to studies directly related to coatings of this type. The final review includes 149 references.

## 2. Classification of Metal Matrix Polymer Composites (MMPCs)

Metal matrix polymer composites (MMPCs) are characterized by a considerable diversity of composition and structure, resulting from the wide range of metallic matrices and polymer fillers, as well as differences in the morphology and distribution of the polymer phase. For the systematic classification of existing MMPCs, it is appropriate to consider three main criteria: the material of the metallic matrix, the type of polymer filler, and its morphology. This approach makes it possible to relate the composition and structure of the composite material to its functional characteristics, which determine its areas of practical application [6,22,23].

### 2.1. Classification of MMPCs by Metallic Matrix Material

**Figure 1:**
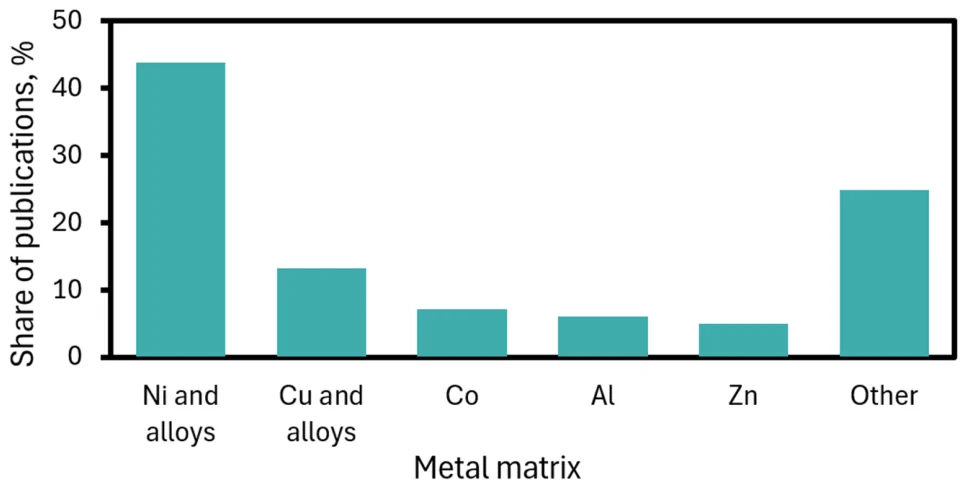
Share of publications by metallic matrix type in composite coatings.

The metallic matrix provides the mechanical strength and corrosion resistance of the coating and its ability to retain polymer particles. Depending on the metallic matrix material, MMPCs can be divided into several main groups (Figure 1): nickel-, copper-, cobalt-, aluminum-, titanium-, zinc-, and chromium-based. The choice of matrix material is determined by the required combination of mechanical strength, corrosion and wear resistance, thermal and electrical conductivity, operating temperature, density, and technological compatibility with the polymer filler. Nickel matrices are the most widely used, whereas the application of other metals is primarily determined by specific requirements for the performance characteristics of the composite material.

As mentioned above, Ni is the most widely used metallic matrix in MMPCs. This is due to the technological advantages of nickel electroplating, a well-established and highly versatile method that allows polymer particles to be co-deposited over a wide range of concentrations. Nickel matrices are characterized by high corrosion resistance, hardness in the range of 200–600 HV, depending on the deposition conditions, and good compatibility with fluoropolymers [24,25]. These advantages are confirmed in the review [24], which systematizes the mechanical properties of electroplated Ni composites and demonstrates their superior performance compared with pure nickel coatings. The practical effectiveness of such systems is illustrated by Ni–W/PTFE coatings produced by pulse current electroplating on low-carbon steel, where a substantial reduction in the wear rate is observed at a PTFE concentration of 15 g/L [26].

Cu is an alternative to nickel and is selected when high thermal and electrical conductivity combined with wear resistance are critically important. Copper-based composites with polymer fillers have been investigated as solid lubricants for mechanical switches [26,27,28,29,30]. Examples of Cu/PTFE systems used in plain bearings have also been reported [30].

Cobalt has proven to be a promising metallic matrix for composite materials intended for operation in highly loaded friction assemblies, where high hardness and heat resistance are critically important. The versatility of cobalt is associated with its compatibility with a wide range of fillers, including sulfides, oxides, and fluorides, as well as with the possibility of using various composite fabrication technologies [31,32,33]. This experience makes it possible to extend the principle of a “strong matrix + functional filler” to metal–polymer systems. The hardness and thermal stability of cobalt can provide a basis for composites in which the load-bearing capacity of the metal is effectively combined with the specific properties of polymer components.

Among other metallic matrices, Cr, Zn, Al, and Ti are also used. Chromium is traditionally used for corrosion and wear protection; however, modification of chromium coatings with polymer fillers is associated with a number of technological difficulties, primarily high internal stresses and poor compatibility with polymers. Consequently, examples of Cr matrices with polymer fillers are practically absent from the available literature [34,35]. Zinc is predominantly used for corrosion protection of steel components, particularly in Zn–Cr alloys [36,37], whereas studies of Zn–polymer composites, such as Zn/PTFE, remain sporadic. Nevertheless, some studies demonstrate the feasibility of co-depositing polymer particles with zinc [38]. Aluminum matrices are used less frequently with polymer fillers than Ni- or Cu-based matrices, which is attributed to the technological difficulty of incorporating thermally unstable polymers into aluminum. Nevertheless, such composites have been reported. For example, Al–PTFE functionally graded materials containing 25–75 wt.% polytetrafluoroethylene were produced by powder metallurgy, with the polymer uniformly distributed along the grain boundaries of aluminum [39]. In [40], an aluminum matrix (pure Al and alloy 7091) was reinforced with particles of thermoplastic PEEK and liquid-crystalline polyester Xydar. After consolidation and extrusion, the polymer particles were elongated into fibers 9–30 μm in length and uniformly distributed throughout the metal, resulting in reduced density and increased specific strength. Another example is Al6063 alloy-based composites containing recycled PET as a filler, produced by stir casting [41]. These examples demonstrate that, despite technological limitations, combining an aluminum matrix with polymer fillers is feasible and may be promising for the development of lightweight metal–polymer composites.

Titanium and its alloys (e.g., Ti–6Al–4V) are used as substrate materials in biomedical and aerospace applications, where polymer coatings such as polycaprolactone, epoxy resins, and conducting polymers (polyaniline, polypyrrole) are applied onto titanium surfaces to improve biocompatibility, corrosion resistance, and drug-delivery functionality [42]. However, true metal matrix composite coatings with a continuous titanium matrix and dispersed polymer filler particles have not been widely reported, primarily due to the technological difficulty of electrodepositing titanium from aqueous electrolytes. Consequently, in the context of MMPC coatings, titanium is classified as a promising but largely unexplored matrix material, and the available polymer–titanium systems should be considered as polymer-on-metal coatings rather than true MMPCs. As for iron, it is practically absent from the scientific literature as a matrix for polymer composites.

Thus, the analysis of metallic matrices used in MMPCs demonstrates their considerable potential for the development of high-performance composite systems. The choice of metallic matrix critically affects the final properties of the material, determining its mechanical characteristics, electrical conductivity, heat resistance, and biocompatibility. At the same time, the technological compatibility of the matrix with the polymer components is an important factor.

### 2.2. Classification of MMPCs by Polymer Filler Type

In modern tribology, considerable attention is devoted to the development of composite materials with polymer fillers that provide a low coefficient of friction and self-lubricating properties [43,44,45,46,47,48,49,50]. The selection of a specific polymer is determined by the service conditions, including operating temperature, chemical environment, and wear resistance requirements [46]. The following classes of polymer fillers are the most widely used:

- Fluoropolymers, represented by polytetrafluoroethylene (PTFE), are the most widely used modifiers. Their incorporation into the structure of a composite material provides a significant reduction in the coefficient of friction, improved antifriction properties, increased wear resistance, and high chemical inertness of the composite [46,47].
- Thermosetting polymers, in particular epoxy resins, are used in metal matrices such as zinc and aluminum. Their incorporation promotes stronger adhesive interactions between the components, increased corrosion resistance, and improved mechanical strength of the composite [9,18].
- Biocompatible polymers, represented by polycaprolactone, are primarily used as coating materials on metallic substrates (e.g., titanium, magnesium alloys) for biomedical applications. The characteristic features of such systems include osteoconductive properties, the possibility of controlled drug delivery, and material biodegradability [51,52,53]. However, PCL has not been demonstrated as a dispersed filler within a continuous metal matrix in MMPC coatings, and its use in the MMPC context remains a prospective direction [42].
- Electrically conductive polymers (polyaniline and polypyrrole), when incorporated as dispersed particles into metal matrices (e.g., nickel), provide the formation of electrically conductive structures, corrosion protection of the surface, and the possibility of modifying surface properties [54,55]. For example, Ni/PANI composite electrodes produced by electrodeposition from a Watts nickel bath containing suspended polyaniline particles demonstrated enhanced electrocatalytic activity and improved surface passivation compared with pure nickel [47,48].

According to their functional purpose, polymer fillers can be divided into three main groups [45]. Antifriction modifiers include PTFE and its derivatives. Protective fillers are represented by epoxy resins, polyaniline, and polypyrrole, which have been demonstrated primarily in nickel- and zinc-matrix composite coatings. The biomedical group includes polycaprolactone-based coatings on metallic substrates (titanium, magnesium alloys), which fall outside the strict MMPC definition and are considered a related but separate category of polymer-on-metal systems, discussed only as a boundary case.

Current trends in the development of MMPCs include the following areas:

- development of multicomponent polymer systems to achieve a synergistic effect [49,50];
- incorporation of nanostructured polymer modifiers [56,57];
- development of polymer materials with tunable performance characteristics [43,44,45,58];
- development of hybrid composites containing organic corrosion inhibitors [59,60].

The study of polymer fillers for MMPCs demonstrates considerable potential for modifying the performance properties of composite materials. A key factor in achieving this potential is the possibility of obtaining a synergistic effect through the combination of different polymers, which makes it possible to precisely tailor the characteristics of the final product. The use of nanostructured modifiers and hybrid composites with corrosion inhibitors is a promising direction, providing improved tribological and mechanical properties, as well as enhanced resistance in aggressive environments.

### 2.3. Classification by Filler Morphology

The morphology of the polymer phase is a determining factor in the lubrication and strengthening mechanisms of MMPCs. Three main morphological forms of polymer fillers can be distinguished:

- Particle form is the most common form of polymer filler. The characteristic particle size ranges from 0.05 to 50 μm. Uniform distribution of the particles within the metallic matrix is achieved during electroplating. The functional role of the particles is to form a self-lubricating tribofilm upon exposure at the friction surface, while the metallic matrix provides the load-bearing capacity of the material [61,62,63]. According to recent studies, particles with a size of up to 1 μm provide an optimal combination of hardness and tribological properties. Examples of practical applications include Ni–PTFE composites with particle sizes of 0.5–5 μm and Ni–W/PTFE systems with concentrations of 5–20 g/L [26].
- Fiber form—characterized by a diameter of 1–10 μm and a length of 10–50 μm. Their primary purpose is to reinforce the composite and improve its fracture resistance. The use of fibers in electroplated coatings is limited by the difficulty of achieving uniform dispersion. In metallic matrices, fibrous fillers are predominantly used in composites produced by powder metallurgy and plasma spraying [64,65,66].
- Microcapsule form is an innovative approach to the development of self-lubricating materials. The capsules contain liquid or solid lubricants that are released upon mechanical rupture of the shell. This approach provides a “self-healing” effect under boundary lubrication conditions. Recent developments have demonstrated the high efficiency of microcapsule systems, including a reduction in the coefficient of friction of up to 81.9%, operation over a wide temperature range, and effectiveness under high contact pressures. Despite the potential of microcapsules, their application in electroplated coatings is limited by technological constraints. The main incorporation methods are thermal spraying and material impregnation [67,68,69]. It is important to note that microcapsules are considered a polymer filler in the strict sense when the capsule shell is embedded in a continuous metal matrix, rather than as a separate layer.

Thus, the morphology of the polymer phase is a key factor determining the functional properties of MMPCs and requires a comprehensive approach when selecting the filler form for specific service conditions.

The systematic classification of MMPCs by metallic matrix material, polymer filler type, and filler morphology reveals that nickel is the predominant matrix material in the literature, owing to the maturity and versatility of nickel electroplating technology and its broad compatibility with fluoropolymer fillers. Among polymer fillers, PTFE is the most widely used, primarily as an antifriction modifier, while epoxy resins and electrically conductive polymers (polyaniline, polypyrrole) serve as protective additives. Particulate morphology is the dominant filler form, providing the best combination of processability and tribological performance, with particles up to 1 μm offering optimal hardness–friction balance. Fiber and microcapsule morphologies are less common and are mainly associated with powder metallurgy and thermal spraying. Notably, several promising directions—such as cobalt and aluminum matrices, biocompatible polycaprolactone fillers, and microcapsule-based self-healing systems—remain largely unrealized in genuine MMPC coating architectures and represent prospective rather than established solutions.

## 3. Main Methods for Producing MMPCs

As follows from Section 2, the fabrication of MMPCs can be carried out using a wide range of technological approaches, such as powder metallurgy, stir casting, consolidation and extrusion, as well as vapor deposition (PVD/CVD). These methods are mainly focused on producing bulk composite materials or serve an auxiliary role in coating fabrication and are therefore beyond the scope of this review.

This section focuses on the methods most widely used specifically for the fabrication of MMPC coatings and offering the greatest potential in terms of technological flexibility and control of functional properties: electroplating, electroless plating, thermal and cold spray, as well as additive and hybrid technologies. A detailed description of these methods is provided below, including an analysis of their advantages, limitations, and fields of application.

### 3.1. Electrodeposition

**Figure 2:**
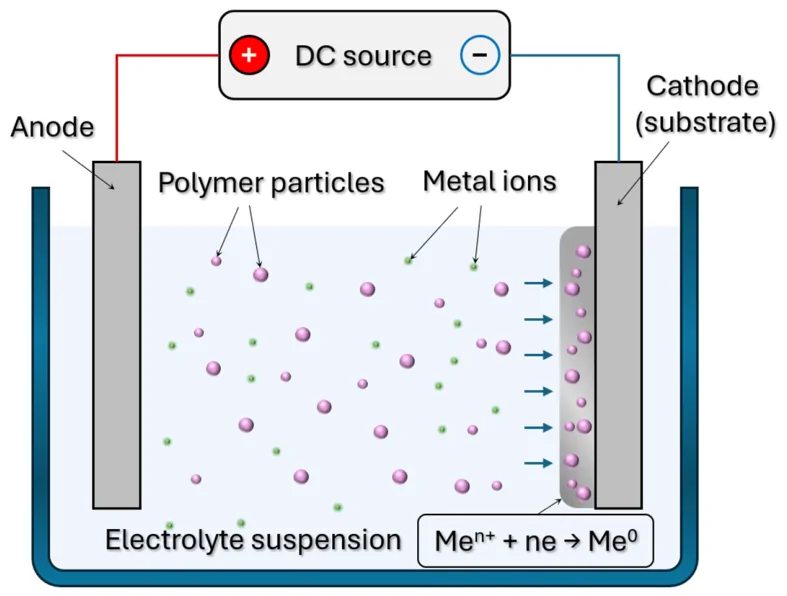
Schematic illustration of the electrodeposition of MMPC coatings.

Electroplating is one of the most widely used methods for fabricating MMPCs due to its technological simplicity, precise control over process parameters, and relatively low processing temperature. The method is based on the co-deposition of the polymer filler and matrix metal from an electrolyte suspension under an external electric field: dispersed polymer particles migrate toward the cathode and become incorporated into the forming metallic matrix (Figure 2).

The key advantages of the method include high coating–substrate adhesion resulting from the direct electrochemical deposition of the metallic phase onto the electrically conductive substrate, as well as precise control over coating thickness and phase composition. It should be emphasized that this advantage concerns the adhesion of the coating to the substrate; it does not extend to the strength of the internal interface between the metallic matrix and the embedded polymer particles, which is governed by the surface chemistry of the filler and is discussed in detail in Section 4.5. In particular, for low-surface-energy fillers such as PTFE, matrix–filler bonding in electrodeposited coatings relies predominantly on mechanical interlocking and weak physical interactions (Section 4.5.2). Suitability for processing products with complex geometries and scalability from laboratory-scale synthesis to industrial production complete the list of advantages: suitability for processing products with complex geometries and scalability from laboratory-scale synthesis to industrial production. At the same time, the method is associated with several technological limitations: the need for careful selection of compatible metal/polymer pairs due to the potential degradation of polymer particles in the aggressive electrolyte environment; the risk of filler agglomeration and, consequently, non-uniform distribution of the dispersed phase at high polymer concentrations; as well as dimensional and technological limitations determined by the size of the electrochemical bath [10,38,70,71,72,73,74].

In recent years, significant progress has been made in the development of MMPC electroplating technology. Thus, a fundamental study devoted to a systematic analysis of the effect of PTFE concentration on the formation and properties of Ni–W/PTFE composite coatings demonstrated that, when the PTFE concentration was varied in the range of 5–20 g/L, the coatings produced at 15 g/L PTFE exhibited the optimal properties. These coatings were characterized by an ultrafine-grained structure with a crystallite size of 9.44 nm and demonstrated the best tribological characteristics, including a minimum coefficient of friction of 0.24 and a wear rate of 1.77 × 10^−7^ mm^3^/(N × m). When the PTFE concentration was increased to 20 g/L, intensification of particle agglomeration was observed, resulting in deterioration of the performance characteristics. A significant contribution to the development of electroplating was made by the study [75], which investigated the effect of pulse-mode parameters on the formation of Ni–P–Al_2_O_3_–PTFE composite coatings. The study demonstrated the possibility of deliberately controlling the structural and mechanical characteristics of the material by modifying the pulse duty cycle, opening prospects for the development of coatings with tailored properties. Recent review studies have made it possible to systematize the key aspects of MMPC electroplating technology. The work [76] provides a comprehensive analysis of technological challenges, including methods for modifying the surface of filler particles, approaches to stabilizing electrolyte suspensions, and the effect of electrolyte composition on the co-deposition kinetics of the components. Particular attention in [25] is devoted to strategies for dispersing solid lubricant nanoparticles (PTFE) in a nickel matrix, which is of fundamental importance for ensuring structural uniformity and reproducibility of the properties of composite materials. These studies demonstrate significant progress in the development of MMPC electroplating technology and open up prospects for the fabrication of materials with improved performance characteristics.

### 3.2. Electroless Plating

**Figure 3:**
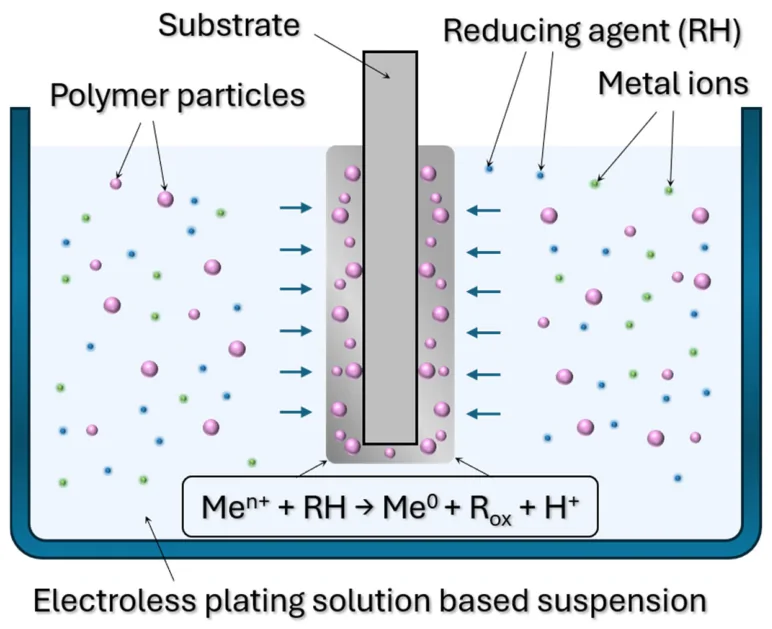
Schematic illustration of the electroless deposition of MMPC coatings. RH is a reducing agent, and R_ox_ is the product of its oxidation.

Electroless plating is a highly efficient technology for fabricating MMPCs with a uniform distribution of components on surfaces with complex topologies without applying an external electrical potential. The process is based on the autocatalytic reduction of metal ions (primarily nickel or copper) with simultaneous incorporation of dispersed polymer particles into the forming metallic matrix (Figure 3).

The advantages of this method include uniform coating distribution on surfaces of any configuration, no need for an external power source, the possibility of depositing composite coatings onto non-metallic substrates (ceramic and polymeric materials) after preliminary activation, and excellent tribological and anticorrosion performance. However, the technology also has several limitations: a relatively low coating deposition rate compared with electroplating, difficulties in maintaining a stable electrolyte composition, a maximum coating thickness of 50 μm, and the need for stabilizing additives and surfactants to prevent aggregation of dispersed particles [77,78,79,80,81,82].

Significant progress in electroless plating technology was demonstrated in study [82], which presented an innovative two-stage approach to the fabrication of Ni–P–PTFE composite coatings. The process consists of two successive treatment stages: the first stage (20 min) involves static immersion of the sample in the plating bath, which ensures effective adsorption of PTFE particles onto the substrate surface; the second stage (20 min) is carried out under intensive agitation of the medium (200 rpm at a temperature of 88 °C). The resulting coating exhibited the following characteristics: the mass fraction of the polymer component (PTFE) in the composite layer reached 25.96%. The deposition rate was 3.44 μm over 40 min; the coefficient of friction was 0.195. The static water contact angle reached 141.9°, indicating pronounced hydrophobic properties of the surface, and the surface energy was 2.74 mN/m. The developed methodology enables the fabrication of submicron-scale coatings (with a thickness of less than 5 μm) with a combination of outstanding surface properties.

The mechanism of composite structure formation involves the following successive stages: convective transport of dispersed polymer particles toward the substrate surface; adsorption of the particles onto the metallic surface through van der Waals interactions and adsorption of surfactant cations; and incorporation of polymer particles during the growth of the metallic matrix. Cationic surfactants, in particular cetyltrimethylammonium bromide (CTAB), are used to optimize the incorporation of the polymer phase into the composite, thereby enhancing the efficiency of composite structure formation [77,80,82,83].

### 3.3. Thermal Spraying

**Figure 4:**
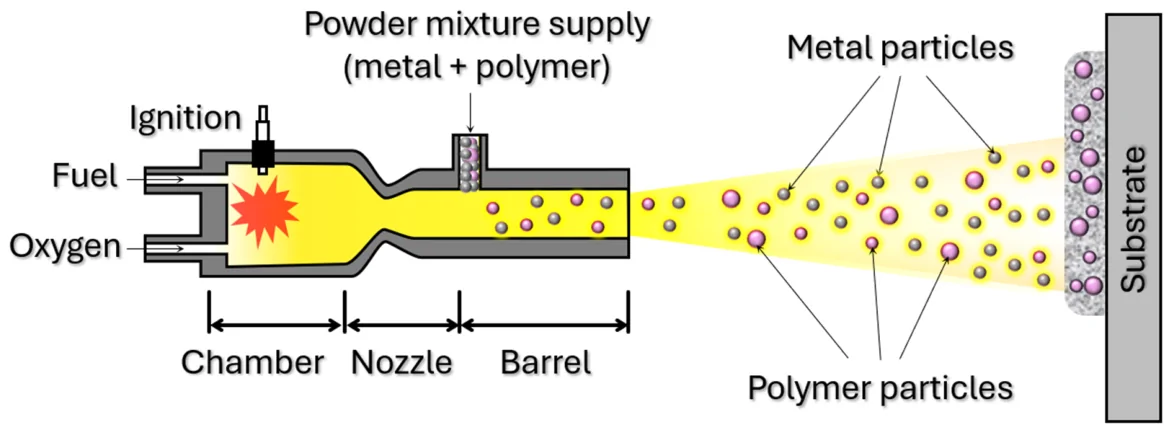
Schematic illustration of the HVOF spraying of MMPC coatings.

Thermal spray (including plasma, flame, and HVOF processes) is a high-temperature technology for fabricating composite coatings based on the melting or partial melting of the feedstock material (powder compositions or wire feedstock), followed by high-velocity transfer of the particles onto the substrate (Figure 4).

The technological features of the method are determined by the extreme temperature conditions of the process (1500–3000 °C), which impose significant limitations on the selection of polymer components due to the risk of thermal degradation. The advantages of the technology include high process productivity, with the possibility of producing coatings up to several millimeters thick; versatility for large-sized products with complex geometries; and a wide range of available spray materials. The limitations of the method include the thermal instability of polymers with melting points below 300–350 °C (including PTFE); the need to use high-temperature-resistant polymer materials such as polyether ether ketone (PEEK) and polyimides; and potential thermal degradation of the polymer phase during spraying [84,85,86,87,88].

Recent advances in thermal spray demonstrate significant progress in the development of technological solutions. For example, study [89] presented an innovative single-stage method for flame spraying PEEK (glass transition temperature of 143 °C, melting point of 343 °C) onto low-carbon steel. The technology is based on the following key parameters: preheating of the substrate; strict control of interpass temperatures during processing; and optimization of the flame flow parameters. The resulting coating demonstrated an adhesion strength of 16 ± 3 MPa, a microhardness of 17 HV (increasing to 29 HV after heat treatment), a coefficient of friction of 0.31 after heat treatment, and a 50% reduction in the wear rate (to 10.5 × 10^−6^ mm^3^/(N × m)). An important study on the thermal spray of polymers presented in [90] demonstrates that controlled-temperature flame spraying is optimal for thermally sensitive polymer composites. In contrast, plasma spraying is characterized by substantial degradation of the polymer phase, which limits its application for the fabrication of composite coatings while preserving the functional properties of the polymer component.

### 3.4. Cold Spray

**Figure 5:**
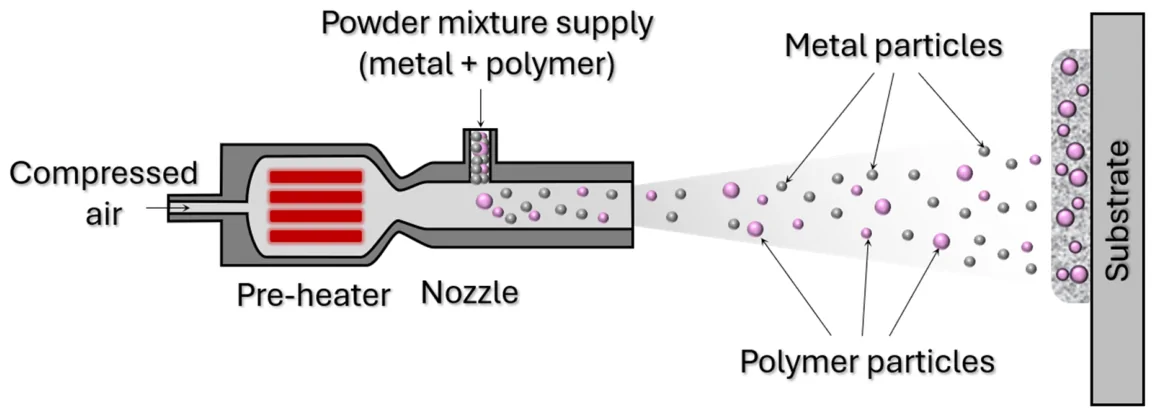
Schematic illustration of the cold spray of MMPC coatings.

Cold spray is a solid-state process for fabricating composite coatings, characterized by supersonic particle velocities (500–1200 m/s) at relatively moderate gas-flow temperatures (up to 600 K). The physical and technological principles of the method are based on the plastic deformation of dispersed particles upon their high-velocity impact with the substrate surface without transition to the molten state (Figure 5).

The main advantages of the technology include preservation of the structural integrity of the polymer phase without thermal degradation; retention of the initial microstructure and physicomechanical properties of the powder material; absence of oxidation during coating formation; and the possibility of deposition onto thermally sensitive materials, including polymer composites and aluminum alloys. The limitations include significant capital investment required for specialized equipment; substantial restrictions imposed by the plastic properties of the sprayed materials, as brittle polymer components are susceptible to fragmentation under impact; and the need to achieve high particle kinetic parameters to form strong interparticle bonds [91,92,93,94,95].

Study [96] demonstrated the successful fabrication of metal–fluoropolymer composite coatings on an St3 steel substrate. The incorporation of ultrafine PTFE into a Cu–Zn matrix substantially improved the corrosion resistance of the material. For a specimen with an intermediate Cu–Zn underlayer subjected to heat treatment at 500 °C, followed by friction treatment with ultrafine PTFE and subsequent annealing at 350 °C, the following parameters were recorded: a corrosion current density of 8.5 × 10^−7^ A/cm^2^ and an impedance modulus of |Z| = 5.3 × 10^4^ Ω × cm^2^, which was 25 times lower than that of the reference Cu–Zn coating. The fluoropolymer component effectively fills structural defects and pores in the metallic matrix, creating an effective barrier against electrolyte penetration. Study [97] presented the results of cold spraying Cu–polyamide (PA6) composite coatings, in which polyamide served as the binder phase for the copper particles; the coatings were deposited onto polymer substrates at a gas temperature of 260 °C and pressures ranging from 0.41 to 1.37 MPa. The copper content in the initial powder mixture varied from 30 to 75 vol.%. Notably, the copper particles retained their spherical morphology upon impact and did not undergo significant plastic deformation; the cohesion of the as-sprayed layer was ensured by the polymeric binder together with mechanical compaction of the powder. Subsequent heat treatment by hot pressing at a temperature below the melting point of PA6 (~220 °C) improved the cohesive strength of the coatings through softening of the polyamide phase and densification of the composite.

### 3.5. Hybrid Methods and Additive Technologies

The concept of hybridization of technological processes represents a promising direction in the development of composite material fabrication methods, making it possible to compensate for the limitations of individual approaches through their rational combination. In the context of MMPC fabrication, hybrid strategies are particularly relevant because none of the methods discussed above can simultaneously ensure strong adhesion of the polymer phase to the metallic matrix, preservation of the polymer structure, and control over filler distribution. The following hybrid approaches are considered, in which a metallic matrix with a polymer filler is formed through the sequential or simultaneous application of two or more technologies.

- Thermal spray of a porous metallic matrix followed by polymer impregnation. This method is a two-stage process in which a porous metallic coating (bronze, nickel, or stainless steel) is first produced by thermal spray (plasma, flame, or HVOF), followed by impregnation of the pores with a polymer composition (PTFE, epoxy, or phenolic resins) [98,99]. The porous metallic structure, containing 10–30 vol.% pores, acts as the load-bearing matrix, while the polymer filling the pores serves as a functional filler that reduces the coefficient of friction and provides chemical resistance. This approach is widely used in the production of self-lubricating plain bearings. For example, in [98], a steel substrate was coated with a porous sintered bronze layer, whose pores were impregnated with a mixture of PTFE and lead. During friction, PTFE gradually emerges from the pores, forming a lubricating film on the contact surface. A similar approach was implemented for thermally sprayed coatings: in [100], porous thermally sprayed metallic coatings were sealed with a PTFE emulsion, while ultrasonic impregnation more than doubled the depth of polymer penetration into the pores compared with conventional impregnation, resulting in reduced porosity and improved corrosion resistance.
- Thermal spray of a composite coating with polymer microcapsules incorporated into the forming metallic matrix. In [67], polyurethane-shell microcapsules containing a liquid lubricant (polyalphaolefin, PAO) were incorporated into a metallic matrix (stainless steel) using arc, plasma, and high-velocity oxy-fuel air spraying (HVOAF). The hybrid nature of the process lies in the two-stage feed: first, a layer of the metallic matrix is sprayed, followed by injection of the microcapsules into the spray plume, where they become embedded in the pores and interparticle spaces of the metallic framework. The use of low-temperature spraying conditions (arc spraying) made it possible to avoid degradation of the polymer capsule shells. The microcapsule-containing coatings provided a consistently low coefficient of friction (<0.2); during wear, the shells ruptured and released the lubricant, thereby implementing a “self-lubricating” effect within the metallic matrix.
- Cold spray of metal–polymer mixtures with a high metal content followed by post-treatment. Cold spray of mixtures of metal and polymer powders with a high metal content (up to 75 vol.% Cu) produces a structure in which the metal particles form a dense interconnected network, while the polymer fills the interparticle spaces and pores, acting as a binder and dispersed filler. In [97], cold spray of a Cu + PA6 (polyamide 6) mixture at a temperature of 260 °C and a pressure of 0.41–1.37 MPa, with copper concentrations of 30, 50, and 75 vol.% in the initial powder, was investigated. Because the polymer particles do not deform plastically upon impact, the cohesion of the as-sprayed layer relies on the polymeric binder; subsequent hot pressing at a temperature below the melting point of PA6 further consolidated the structure through softening of the polyamide within the interparticle spaces. After hot pressing, the copper content in the coating reached 67–76%, reflecting the consolidation of the metallic framework and the partial displacement of the polymer phase to the interparticle boundaries.
- Ultrasonic additive manufacturing (UAM) with polymer interlayers. UAM is a solid-state additive process in which metal foils (Al, Cu, Ti) are joined layer by layer under ultrasonic vibrations at temperatures not exceeding half the melting temperature of the matrix metal. The low process temperature makes it possible to incorporate thermally sensitive polymer materials directly into a metallic matrix without degradation. In [101], polymer films, in particular polyvinylidene fluoride (PVDF), were incorporated into an aluminum matrix between foil layers. During ultrasonic welding, plastic flow of the metal envelops the polymer inclusions, forming a strong mechanical bond. Similarly, in [102], NiTi and optical fibers were successfully incorporated into an aluminum matrix by UAM at temperatures below 200 °C, confirming the applicability of the method for fabricating layered metal-matrix composites with polymer fillers.

Directed Energy Deposition (DED) remains at the experimental research stage for applications involving MMPCs. Current developments are mainly focused on the fabrication of functionally graded coatings with ceramic fillers, in which the polymer component is not involved. UAM, described above, appears more promising for MMPCs because its low-temperature solid-state processing is compatible with thermally sensitive polymers. Hybrid technologies have considerable potential, as they make it possible to compensate for the limitations of individual methods, achieve complex functional properties, provide more reliable adhesion between the components, and more precisely control microstructure formation. At the same time, implementation of such approaches is associated with a number of challenges: the technological process is highly complex, requires careful synchronization of the parameters of different methods, imposes increased demands on equipment, and is not always readily scalable. In the future, development of this field will involve improving existing hybrid solutions and creating new combinations of technological methods, making it possible to fabricate MMPCs with predefined characteristics while maintaining acceptable manufacturing processability.

A comparative analysis of MMPC fabrication methods is a key aspect in selecting an appropriate technology for producing materials with tailored properties. To clearly demonstrate the advantages and limitations of different approaches to MMPC fabrication, Table 1 summarizes the main characteristics of the existing methods.

Among the fabrication technologies considered, electrodeposition is the predominantly used method in the literature, which is attributed to its technological simplicity, low processing temperature (40–60 °C), precise control over coating thickness and composition, and scalability from laboratory to industrial production. Electroless plating extends the capabilities to complex-shaped components and non-metallic substrates but is limited by a low deposition rate and a maximum coating thickness of ~50 μm. Thermal spraying offers high productivity and thick coatings but is constrained by the thermal degradation of most polymers, restricting filler selection to heat-resistant polymers such as PEEK and polyimides. Cold spray preserves the integrity of the polymer phase but requires significant capital investment. Hybrid methods are identified as the most promising direction, as they can compensate for the limitations of individual techniques and enable the fabrication of coatings with tailored architectures; however, their practical implementation is complicated by the need for precise process synchronization and remains difficult to scale.

## 4. MMPC Structure Formation

The formation of the MMPC structure is determined by the deposition method (electroplating, electroless plating, thermal spray, cold spray, etc.). Regardless of the method, the key factors include the mechanism of polymer particle incorporation (adsorption, mechanical anchoring, entrapment from the melt), particle size and concentration, particle dispersion conditions, and the nature of the interfacial boundary. The structure of MMPCs produced by different methods may differ substantially in terms of filler distribution uniformity and interfacial bonding strength, which directly affects the tribological and mechanical properties of the resulting MMPC coatings.

### 4.1. Mechanisms of Polymer Particle Incorporation into the Metallic Matrix

In modern technologies for MMPC fabrication, the mechanism of polymer particle incorporation into the metallic matrix plays a key role. The most common method is electroplating, in which a sequence of interconnected processes takes place. At the first stage, dispersed particles are transported toward the cathode surface. This process occurs through two main mechanisms: convective transport during agitation and electrophoretic motion activated by the presence of a charge on the particles. The efficiency of particle transport depends substantially on the selected deposition technology and electrolyte parameters. The next stage is particle adsorption onto the growing metallic surface. This process is governed by a combination of interactions involving van der Waals forces and the effect of surfactants. Cationic surfactants, such as cetyltrimethylammonium bromide, play a particularly important role, as they not only stabilize the suspension but also significantly increase the efficiency of particle incorporation into the coating structure. The final stage is particle incorporation, during which polymer inclusions become embedded in the growing metallic layer. The formation of a strong interfacial bond is ensured by irreversible adsorption, which requires overcoming the desolvation energy barrier of both the polymer particle surface and the metallic substrate surface [38,76,103,104].

During electroless plating, polymer particles are incorporated into the growing metallic layer through adsorption onto the activated substrate surface followed by the reduction of metal ions from the solution. The process does not require an external current. Here, hydrodynamic agitation becomes a key factor, both external (mechanical rotation, bubbling, and ultrasonic treatment) and that generated by gas evolution (hydrogen) in the near-surface region, together with the adsorption capacity of the particles [77,105,106].

During thermal spray (including plasma and high-velocity processes), the use of polymer particles as fillers in a metallic matrix is substantially limited by the high temperatures in the spray plume. Under these conditions, polymers tend to undergo partial melting or thermal decomposition, resulting in the loss of functional properties and the formation of undesirable by-products. Therefore, the polymer phase is generally introduced not as a free filler but in a modified form (either as a coating on metal powder particles or as a separate component of pseudo-alloy powders). In this context, pseudo-alloy powders are not true alloys but specially prepared powder compositions in which the components retain their phase identity. In particular, the polymer may be present as a mechanical mixture with the metal powder or in the form of agglomerated particles (granules), in which the polymer and metallic components are combined through adhesion or a temporary binder without alloying. During spraying, such compositions disintegrate in the high-temperature flow, while the polymer is retained in the coating as a discrete phase, providing, for example, a reduction in the coefficient of friction. At the same time, the fraction of polymer that retains its original structure in the final coating is usually small and is determined by the balance between the particle residence time in the spray plume and the thermal stability of the polymer [90,107,108].

In cold spray, metallic particles become embedded in the substrate and in one another through plastic deformation at high velocities (600–1200 m/s). Polymer particles, which are softer and typically deform less upon impact, can become anchored in the pores and crevices of the forming layer. Thus, study [97] demonstrated that cold spraying of a copper–polyamide mixture yields a composite coating in which the copper particles are held together and retained by the polymeric binder occupying the interparticle spaces; additional anchoring is provided by mechanical trapping within the deposited layer and at the substrate surface.

When hybrid methods are used for MMPC fabrication, the mechanisms of polymer particle incorporation into the metallic matrix are multistage and depend on the specific combination of technologies employed (see Section 3.5). However, the following key mechanisms can be identified: capillary impregnation (spraying + impregnation) [98,99,100], mechanical trapping in pores (microcapsule spraying) [67], plastic flow of metal around the particles (UAM) [101], mechanical interlocking followed by autocatalytic metal growth (cold spray + electroless plating) [102], and combined adhesion through an intermediate layer (PVD/CVD + hot pressing). In all cases, the interfacial bonding is predominantly mechanical, while the formation of chemical bonds between the polymer and metal is unlikely in low-temperature hybrid processes.

Thus, the mechanisms of polymer particle incorporation into the metallic matrix are determined by the selected MMPC fabrication method and the combination of technological process parameters. The efficiency of filler incorporation depends on particle size, surface activity, hydrodynamic conditions, and the nature of interfacial interactions. At the same time, the formation of a strong interfacial bond is a key factor ensuring the retention of the functional properties of the polymer component within the MMPC.

### 4.2. Effect of Filler Particle Size

The size of polymer particles has a substantial effect on their incorporation into the coating, distribution within the matrix, and the resulting strengthening effect; this relationship manifests differently depending on the method used to fabricate the composite material.

During electroplating, nanoscale particles (50–500 nm) provide the most uniform distribution and maximum strengthening, which is attributed to dispersion strengthening and grain refinement of the matrix. According to the review [25], this particle size range is optimal for nickel composites with PTFE. The effectiveness of nanoparticles was experimentally confirmed, where the use of PTFE with an average particle size of 260 nm resulted in a minimum wear rate of 1.77 × 10^−7^ mm^3^/(N × m). At the same time, larger particles (in the micrometer range) tend to settle and agglomerate, which may lead to the formation of defects in the coating.

During electroless plating, the size of polymer particles plays a role no less important than during electroplating. For Ni–P–PTFE MMPC coatings, the submicron range of 0.2–0.5 μm is considered optimal. This filler particle size ensures uniform polymer distribution and prevents agglomeration, which deteriorates the morphology and reduces the mechanical properties of the coating. This is confirmed by a number of studies. In [82], the use of a PTFE dispersion made it possible to achieve a polymer content of up to 25.96% with a uniform particle distribution. In [109], for Ni–P + PTFE duplex coatings intended for geothermal applications, an average PTFE particle size of ~307 nm ensured homogeneity throughout the entire layer thickness (~20 μm). Study [110] showed that at a PTFE concentration of 5.0 g/L in the bath and a particle size of 0.3 μm, the polymer volume fraction reached 27.6%. It is also important to relate the size of the polymer filler particles to the coating thickness: the maximum thickness of electrolessly deposited layers generally does not exceed 50 μm [77,78,79,80,81,82]. As shown in the review [111], incorporation of filler particles approximately 10 μm in size into coatings less than 10 μm thick results in unsatisfactory deposit quality and structural weakening.

During thermal spray, incorporation of polymer particles 10–50 μm in size into the coating is technically possible; however, their thermal instability substantially limits the practical applicability of this method. According to recent studies, heat-resistant polymers (PEEK and polyimides) are the most suitable for thermal spray, whereas less thermally stable polymers (e.g., PTFE) undergo significant degradation [87,90,92]. In cold spray, filler particle size critically affects the efficiency of their capture by the metallic matrix. Studies suggest that filler particles of several micrometres (5–10 μm) are incorporated more efficiently than submicron particles owing to their higher kinetic energy, which facilitates their anchoring in the deformed metallic matrix [91,92,93,112,113].

When hybrid methods are used to fabricate MMPC coatings, the optimal size of the polymer component varies from submicron particles (0.2–0.5 μm) in impregnation emulsions to films 25–100 μm thick in the UAM process and microcapsules 30–70 μm in diameter during thermal spray. The size parameters of the polymer phase should be selected with consideration of the architecture of the composite being formed, the dimensional characteristics of the pore space or interlayer gaps in the metallic matrix, and the temperature limitations of the specific combination of fabrication methods [84,90,101,102,108].

The analysis of the effect of filler particle size on MMPC structure formation shows that the optimal particle size varies depending on the material fabrication method and the required service characteristics. For electroplating, nanoscale particles (50–500 nm) are preferred, as they provide maximum strengthening and uniform distribution. For electroless plating, the submicron range (0.2–0.5 μm) is optimal, whereas for thermal spray, particles 10–50 μm in size are acceptable, provided that their thermal stability is taken into account.

### 4.3. Effect of Filler Concentration on Structure

The concentration of the polymer component is one of the key technological parameters determining the structural characteristics of the MMPC coatings being formed. Unlike particle size and incorporation mechanisms, discussed in Section 4.1 and Section 4.2, the filler concentration in the initial medium (electrolyte, plating bath, powder mixture, or gas stream) directly controls the volume fraction of the phases in the composite, the uniformity of their distribution, and, in some cases, the architecture of the material itself. The effects of concentration vary substantially depending on the MMPC fabrication method.

During electroplating, the kinetics of co-deposition of polymer particles with the metallic matrix can be described by an irreversible adsorption model, according to which an increase in particle concentration in the electrolyte leads to an increase in the filler volume fraction in the coating only until surface saturation is reached, after which a further increase in concentration does not result in a proportional increase in the polymer content [104]. Exceeding the optimal concentration range causes particle agglomeration, accompanied by a decrease in the deposition rate, an increase in porosity, and deterioration of the mechanical and tribological characteristics of the coating [24]. For nickel composites with fluoropolymer fillers, the working concentration range is generally limited to values that ensure homogeneous particle distribution without coagulation; the specific optimum depends on the type of polymer, particle size, and deposition conditions [76]. The use of pulsed current makes it possible to deliberately control the content of the polymer phase by modifying the pulse duty cycle, thereby expanding the achievable range of filler concentrations while maintaining structural uniformity [75,76].

During electroless plating, the concentration of polymer particles in the solution determines the filler mass fraction and the uniformity of its distribution throughout the coating thickness. Since particle transport to the surface occurs hydrodynamically rather than through an electric field, the efficiency of polymer incorporation depends on the suspension concentration and agitation conditions. A two-stage approach to the fabrication of Ni–P–PTFE coatings, involving an initial static immersion stage for adsorption of PTFE particles onto the substrate followed by deposition under intensive agitation, makes it possible to achieve a high polymer content while maintaining its uniform distribution throughout the layer cross-section [82]. The type of surfactant used to stabilize the suspension also has a substantial effect on the efficiency of polymer incorporation at a given concentration and, consequently, on the structure and corrosion properties of the resulting composite [83].

During thermal spray, the concentration of the polymer component in the initial powder composition is limited by the risk of thermal degradation: at high polymer concentrations, the fraction of degraded particles increases, resulting in the loss of functional properties and the formation of undesirable by-products [84,90]. Therefore, the polymer phase is often introduced not as a free filler, but in a modified form—as a component of pseudo-alloy powder compositions or as a coating on metal powder particles—which makes it possible to reduce the effective polymer concentration in the high-temperature spray plume and thereby decrease the degree of its degradation [107,108]. The fraction of polymer retaining its original structure in the final coating is determined by the balance between its initial concentration in the powder mixture, the particle residence time in the high-temperature flow, and the thermal stability of the polymer [90]. When microcapsules are used, their concentration in the flow determines the frequency of their capture by the metallic matrix and, consequently, the effectiveness of self-lubrication upon subsequent mechanical rupture of the shell [66,67].

In cold spray, the concentration of polymer powder in the gas stream directly affects the fraction of particles captured by the matrix. For polymer fillers, systematic data on the effect of concentration on deposition efficiency remain limited. In cold spraying of metal–polymer mixtures with a high metal content, the concentration of the polymer component in the initial powder composition determines its volume fraction in the coating. The metal particles form a continuous interconnected framework, while the polymer phase is localized primarily in pores and at interparticle boundaries of the deformed metallic matrix; subsequent hot pressing can further consolidate the structure, increasing the relative content of the metallic phase in the coating to 67–76% [97]. The fluoropolymer content in cold-sprayed composite coatings also depends on both its concentration in the initial mixture and the gas-flow parameters [96].

When hybrid methods are used, the concentration of the polymer phase is determined not by the composition of the suspension or powder mixture but by the architecture of the composite being formed and the specific combination of technologies employed. In the case of porous metallic coatings followed by polymer impregnation, the concentration of the polymer phase is determined by the porosity of the metallic matrix and the density of the impregnation composition. For example, the pores of a sintered bronze matrix can be filled with a PTFE–lead mixture [98] or used as a sealing medium to improve wear and corrosion resistance [100]. In UAM, the concentration of the polymer component is determined by the thickness of the polymer films and the frequency of their placement between the metallic foil layers, while plastic flow of the metal during welding envelops the polymer inclusions, forming a layered architecture with a predetermined phase ratio [101,102].

Thus, for each of the MMPC fabrication methods considered, there is an optimal concentration range of the polymer phase, exceeding which leads to particle agglomeration, reduced uniformity of filler distribution, and deterioration of the service characteristics. The optimum concentration should be selected with consideration of the specific combination of matrix and filler materials, the fabrication method employed, the required composite architecture, and the target set of coating properties.

### 4.4. Filler Distribution

The distribution of polymer particles throughout the volume and thickness of the coating may be either homogeneous or heterogeneous, depending on the method used to fabricate the composite and the selected process parameters.

The distribution of polymer particles during electroplating may be either homogeneous or heterogeneous and is directly determined by the process parameters and electrolyte composition. Uniform particle distribution can be achieved under optimized conditions, including intensive agitation (100–200 rpm), the addition of surfactants, and careful control of current density. Hydrodynamic conditions in the cathode region play a critically important role in this process. According to the conclusions of [73], they largely determine the uniformity of particle incorporation. Thus, scanning electron microscopy (SEM) and EDX mapping confirmed a homogeneous distribution of PTFE in the Ni–W matrix at a current density of 10 A/dm^2^. This uniformity correlated with the minimum coefficient of friction. At the same time, an increased filler concentration or insufficient agitation causes particle segregation, manifested as enrichment of the near-surface layer of the coating. For the Ni–PTFE system, uniform distribution of PTFE throughout the entire coating thickness (approximately 60 μm) was achieved in [112]. Data obtained using a focused ion beam demonstrated the absence of large agglomerates and the uniform distribution of PTFE particles in the nanocrystalline nickel matrix. Elemental distribution maps additionally confirmed the homogeneity of the structure. An important functional consequence of such uniformity is the “self-recovery” of superhydrophobic properties during abrasion, enabled by continuous renewal of the surface during wear.

The distribution of filler particles in composite coatings fabricated by electroless plating is determined by a combination of technological and physicochemical factors. In particular, for electrolessly deposited Ni–P–PTFE coatings, suspension stability is critical, as it directly affects the uniformity of particle incorporation. As shown in [82], the use of a two-stage process involving preliminary dispersion of PTFE in the plating bath followed by intensive agitation makes it possible to achieve homogeneous particle distribution throughout the coating thickness. More generally, during electroless plating, filler distribution is also affected by the solution agitation rate and the characteristics of particle adsorption onto the substrate surface, highlighting the need for comprehensive control of both hydrodynamic and interfacial process parameters [83].

Under thermal spray conditions, the direct incorporation of polymers into the powder mixture is substantially limited by their thermal instability at temperatures of 1500–3000 °C. To overcome this limitation, the polymer phase is introduced in modified forms. The most common approach involves pseudo-alloy compositions, in which polymer and metallic components are combined through adhesion or a temporary binder. Another effective approach is to apply the polymer as a coating onto metal particles, which helps preserve the polymer phase at high temperatures. Microcapsules with protective shells are also of particular importance, as they can retain their integrity during spraying. Their distribution within the metallic matrix is determined by their concentration in the spray stream, size (typically 30–70 μm), temperature conditions, and feed rate [84,89,90].

During cold spray, the distribution of polymer particles is often heterogeneous. Due to their relatively low hardness, polymers tend to concentrate at interparticle boundaries and in pores formed between deformed metallic particles. Thus, study [97] demonstrated that in Cu–PA6 coatings the polyamide phase is localized primarily at the boundaries between copper particles, acting as a binder, while copper forms the load-bearing framework. At the same time, the distribution may differ in individual systems. For example, SEM images of Cu–Zn coatings with PTFE show uniformly distributed dark polymer regions. Such visual uniformity reflects the characteristics of the specific composition and spraying conditions. Subsequent heat treatment also affects the distribution and retention of the polymer: annealing at 500 °C results in the formation of needle-like oxide crystallites that create a developed surface relief, promoting mechanical interlocking of the polymer and potentially altering its local distribution within the coating structure [96].

Hybrid methods demonstrate considerable potential. They are based on the use of porous metallic matrices that are impregnated with a polymer after spraying. In this case, the polymer fills 10–30 vol.% of the pores of the metallic substrate. Microcapsule-based compositions provide a discrete distribution of polymer inclusions, while cold spray technologies make it possible to obtain materials in which the polymer phase is localized primarily in the pores and at the interparticle boundaries of the deformed metallic matrix. At a high initial metal content (up to 75 vol.% Cu), the metal particles form a dense interconnected framework, while the polymer acts as a dispersed filler distributed within the interparticle spaces; subsequent hot pressing at a temperature below the polymer melting point further consolidates the structure and increases the relative copper content in the coating to 67–76% [97]. In UAM, polymer components in the form of films 25–100 μm thick or fibers are distributed between layers of metallic foil. Plastic flow of the metal during welding ensures reliable encapsulation of the polymer inclusions [66,67,98,99,100,101,102].

### 4.5. Metal/Polymer Interface

The state of the metal/polymer interface is a determining factor for the mechanical and tribological characteristics of MMPCs, and its features depend substantially on the method used to fabricate the coating. The nature of the interfacial bonding between the metallic matrix and the polymer filler governs the load transfer efficiency, the resistance to delamination, and the long-term durability of the composite coating under operational conditions. Different fabrication methods are characterized by different types of bonding, which can be classified into four fundamental mechanisms, described in detail below.

#### 4.5.1. Adhesion Mechanisms at the Metal/Polymer Interface

##### Mechanical Interlocking

This mechanism dominates in cold spray, thermal spray, and hybrid methods involving impregnation of porous structures or ultrasonic additive manufacturing (UAM). During high-velocity particle impact (typically 500–1200 m/s in cold spray), metallic particles undergo severe plastic deformation upon collision with the substrate, creating an extensively developed contact surface. The polymer inclusions become anchored in the pores, crevices, and asperities of the deformed metallic matrix, forming a mechanical interlock that does not require chemical bonding between the phases [91,96,97,101,102,113,114,115]. The efficiency of mechanical interlocking depends on several key factors: (i) the surface morphology of the substrate and the metallic matrix, including roughness, porosity, and the presence of micro- or nanoscale asperities; (ii) the plasticity of the metal, which determines the degree of conformal wrapping around polymer particles; (iii) the kinetic energy of the particles, which controls the extent of deformation and the resulting contact area; and (iv) the particle size ratio between the metal and the polymer phase. Study [97] demonstrated that in the Cu–polyamide (PA6) system produced by cold spray at 260 °C, sufficient cohesion was achieved without plastic deformation of the copper particles, which retained their spherical morphology; bonding was provided by the polymeric binder and by mechanical interlocking of the particles within the deposited layer. In hybrid methods using UAM, plastic flow of the metal during ultrasonic welding at temperatures below 200 °C ensures reliable encapsulation of polymer films between metallic foil layers [101,102]. While mechanical interlocking provides adequate bonding for many applications, it is inherently weaker than chemical bonding and may be insufficient under conditions involving high shear stresses, thermal cycling, or aggressive chemical environments.

##### Adsorption Bonding

This mechanism is characteristic of electroplating, electroless plating, and hybrid methods involving ultrasonic impregnation. Polymer particles are retained at the metal surface by van der Waals forces and electrostatic interactions. The energy of van der Waals interactions for a metal–polymer system typically ranges from 0.05 to 0.5 kJ/mol per contact point, which is sufficient for particle retention during the deposition process but significantly weaker than covalent or ionic bonds [38,76,104]. The adsorption process involves overcoming the desolvation energy barrier of both the polymer particle surface and the metallic substrate surface, after which the particle becomes irreversibly incorporated into the growing metallic layer [104]. Cationic surfactants, in particular cetyltrimethylammonium bromide (CTAB), play a crucial role in enhancing adsorption bonding. CTAB adsorbs onto the hydrophobic surface of polymer particles (e.g., PTFE), imparting a positive charge that strengthens electrostatic attraction to the cathode during electroplating and facilitates particle incorporation into the growing metallic layer [77,80,82,83]. In electroless plating, where no external electric field is applied, adsorption is driven by hydrodynamic transport and the intrinsic adsorption capacity of the particles onto the activated substrate surface [77,105,106]. While adsorption bonding ensures effective particle incorporation during coating formation, the resulting interfacial strength is limited by the relatively low energy of van der Waals and electrostatic interactions, making the interface susceptible to degradation under mechanical or thermal stress. It is important to distinguish between the coating–substrate interface and the matrix–filler interface: the electrochemical nature of electrodeposition ensures strong bonding of the growing metallic layer to the conductive substrate, whereas the bonding between the metallic matrix and the embedded polymer particles is formed by particle adsorption and subsequent physical contact, and therefore inherits the limitations of weak interfacial interactions discussed above.

##### Hydrogen Bonding

Hydrogen bonds can form between functional groups of the polymer (–OH, –COOH, –NH_2_, –C=O) and hydroxyl groups on the oxide layer of the metal surface. The energy of a single hydrogen bond ranges from 10 to 40 kJ/mol, which is substantially higher than van der Waals interactions but considerably lower than covalent bonds. The formation of hydrogen bonds requires the presence of polar functional groups on the polymer surface, which can interact with the hydroxylated metal oxide layer [116,117,118,119,120]. For polar polymers such as epoxy resins, polyamides, and polyimides, hydrogen bonding can contribute significantly to interfacial adhesion. However, for non-polar polymers such as PTFE, polyethylene (PE), and polypropylene (PP), the formation of hydrogen bonds is impossible without preliminary surface functionalization. This is a fundamental limitation of fluoropolymer-containing MMPCs, where the extremely low surface energy of PTFE (~16 mN/m) prevents both hydrogen bonding and effective wetting by the metallic matrix. The importance of hydrogen bonding at the metal–polymer interface has been demonstrated in study [116], which showed that the chemical functionality of the polymer surface plays a decisive role in adhesion to aluminum: the introduction of polar groups onto the polypropylene surface increased the interfacial fracture energy by a factor of 3–5 compared with unmodified polypropylene.

##### Chemical (Covalent) Bonding

Chemical bonding provides the strongest type of interfacial interaction, with bond energies in the range of 200–800 kJ/mol. The formation of covalent bonds between the metal and the polymer can occur through several pathways: (i) in high-temperature processes such as thermal spray, where the molten or partially molten polymer can react with the oxide layer of the metal, forming metal–oxygen–carbon or metal–nitrogen–carbon bonds; (ii) through intermediate coupling agents such as organofunctional silanes, diazonium salts, or polydopamine, which form covalent bridges between the metal oxide surface and the polymer matrix; and (iii) when the polymer contains reactive functional groups capable of direct bond formation with the metal surface [116,117,118,120]. In low-temperature methods (electroplating, electroless plating, cold spray), the formation of direct covalent bonds between the metal and the polymer is unlikely without surface modification. Study [117] demonstrated that chemical reactions at the polymer–metal interface can significantly enhance adhesion, with the formation of interfacial chemical bonds confirmed by X-ray photoelectron spectroscopy (XPS) and Fourier-transform infrared spectroscopy (FTIR). The nature and density of the chemical bonds at the interface determine not only the initial adhesion strength but also the resistance of the coating to environmental degradation, particularly in the presence of moisture, which can disrupt weaker interfacial bonds.

The four adhesion mechanisms described above differ fundamentally in their energetic nature, the conditions required for their formation, and the resulting interfacial strength. In practice, multiple mechanisms often operate simultaneously at the metal/polymer interface; however, the dominant mechanism is primarily determined by the fabrication method and the intrinsic properties of the metal–polymer pair. Table 2 provides a systematic comparison of these mechanisms, summarizing their characteristic bond energies, the processing methods with which they are most commonly associated, and the key references supporting each category.

The bond energies listed should be interpreted as approximate ranges for individual interfacial contacts, as the macroscopic adhesion strength of a coating depends on the density of interfacial bonds, the contact area, and the presence of defects such as pores or nanocracks. Notably, the transition from weak physical interactions (van der Waals forces, < 0.5 kJ/mol) to strong chemical bonds (200–800 kJ/mol) spans more than three orders of magnitude in energy, underscoring the critical importance of surface treatment and polymer functionalization strategies for achieving durable metal/polymer interfaces, particularly when low-surface-energy polymers such as PTFE are employed.

#### 4.5.2. Surface Treatment Strategies for Enhanced Adhesion

##### Surfactant Modification of Polymer Particles

Cationic surfactants, primarily CTAB (cetyltrimethylammonium bromide), are widely used in electroplating and electroless plating for two purposes: stabilization of the polymer particle suspension and enhancement of particle incorporation efficiency into the metallic matrix. CTAB adsorbs onto the hydrophobic surface of PTFE particles through its hydrophobic tail, while the positively charged quaternary ammonium head group faces outward, imparting a net positive charge to the particle surface. This surface charge modification serves multiple functions: (i) it prevents particle agglomeration through electrostatic repulsion between similarly charged particles; (ii) it improves the wettability of the hydrophobic PTFE surface by the aqueous electrolyte; and (iii) it enhances the electrostatic attraction between the positively charged particles and the cathode surface during electroplating, facilitating their incorporation into the growing metallic layer [25,76,77,80,82,83,119]. The use of CTAB in the fabrication of Ni–P–PTFE coatings by electroless plating was shown to increase the polymer content in the composite to 25.96% while maintaining uniform particle distribution throughout the coating thickness [82]. However, the surfactant-mediated improvement in adhesion is primarily physical in nature, as CTAB does not form chemical bonds between the polymer and the metal. Consequently, while surfactant modification enhances particle incorporation and distribution, it does not fundamentally resolve the issue of weak interfacial bonding at the metal/PTFE interface.

##### Plasma Treatment

Plasma treatment is applied to modify the surface of both polymer filler particles and metallic substrates. When PTFE is treated with plasma, defluorination of the surface occurs: fluorine atoms are replaced by hydroxyl (–OH), carbonyl (–C=O), carboxyl (–COOH), and other polar functional groups. This modification significantly increases the surface energy from approximately 16 mN/m (untreated PTFE) to 50–62 mN/m (plasma-treated PTFE) and reduces the water contact angle from approximately 115° to approximately 60° [119,120]. The type of plasma gas strongly influences the treatment efficiency. Ammonia (NH_3_) and hydrogen plasmas have been shown to be particularly effective for PTFE: ammonia plasma treatment increases the surface energy to 48.8 mN/m, while the pull-off strength of an aluminum rod bonded to the treated PTFE surface increases from 31 N (untreated) to approximately 200 N (treated) [119]. For hydrocarbon-based polymers such as PE, PP, and PET, oxygen plasma introduces polar groups (–COOH, –OH, –C=O) capable of forming hydrogen bonds with the metal oxide layer or reacting with adhesives [118,120]. However, prolonged plasma exposure can lead to degradation of the polymer surface layer and chain scission, which weakens the near-surface region. Therefore, optimization of treatment parameters (power, duration, gas composition, pressure) is critical to maximize surface functionalization while minimizing structural damage [119,120]. In the context of MMPC coatings, plasma treatment of polymer filler particles before their incorporation into the metallic matrix is a promising strategy for enhancing interfacial adhesion, particularly for fluoropolymers.

##### Silane Coupling Agents

Organofunctional silanes are bifunctional molecules that serve as molecular bridges between the metal oxide surface and the polymer matrix. One end of the silane molecule (the alkoxysilane group, e.g., –Si(OCH_3_)_3_ or –Si(OC_2_H_5_)_3_) hydrolyzes and condenses with the hydroxyl groups on the metal oxide surface, forming stable covalent Si–O–Metal bonds. The other end (an aminopropyl, epoxy, vinyl, or methacryl group) can react with or interpenetrate the polymer matrix, establishing covalent or strong secondary bonds. For example, (3-aminopropyl)triethoxysilane (APTES) and 3-glycidyloxypropyltrimethoxysilane (GPTMS) significantly improve adhesion at the steel–polyamide interface: the lap shear strength increases to 11.9 MPa with APTES treatment compared with approximately 3 MPa without treatment, and the work of fracture increases from 310 to 1570 J/m^3^ [119,120]. Silane layers with thicknesses below 20 nm preserve the integrity of the metal surface while simultaneously providing chemical coupling with the polymer and improving the moisture resistance of the joint [120]. The use of silanes is particularly effective for polar polymers (epoxy resins, polyamides, polyimides) where the organofunctional group can react directly with the polymer. For non-polar polymers such as PTFE and PP, preliminary plasma or chemical activation is required before silane application, since the silane organofunctional group cannot form effective bonds with an inert polymer surface [118,119,120]. In MMPC coatings, silane treatment of either the metallic substrate or the polymer filler particles represents a viable strategy for significantly enhancing interfacial adhesion, particularly in systems where chemical bonding is otherwise unattainable.

##### Chemical Etching

Sodium–naphthalene etching is a classical method for modifying the PTFE surface. The treatment causes defluorination of the PTFE surface, reducing the F/C ratio from 2.0 to 0.2 or below, and introducing hydroxyl, carbonyl, and other oxygen-containing functional groups capable of forming hydrogen bonds. The surface energy increases from 16.4 to 62.2 mN/m, and the water contact angle decreases from approximately 115° to approximately 60° [119]. An important observation from adhesion testing is that failure in sodium–naphthalene-treated PTFE joints occurs cohesively—within the modified PTFE layer—rather than adhesively at the interface, which indicates that the chemical bonds formed at the interface are stronger than the modified near-surface layer of PTFE itself [119]. While this method is highly effective for surface activation, its application to MMPC coatings is limited by the aggressive nature of the etching solution and the need for careful control of treatment depth. Nevertheless, chemical etching remains a benchmark technique for evaluating the maximum achievable adhesion strength with PTFE surfaces.

#### 4.5.3. Interfacial Characteristics in Specific MMPC Systems

During electroplating, interfacial interactions, particularly in the case of fluoropolymers such as PTFE, are often weak due to the low surface energy of PTFE. In such systems, adhesion is predominantly provided by mechanical interlocking. This statement refers specifically to the matrix–filler interface and should not be confused with the high coating–substrate adhesion characteristic of electrodeposition (see Section 3.1): the latter results from electrochemical growth of the metallic layer on the substrate, whereas the former is limited by the weak physical interactions inherent to fluoropolymers (Section 4.5.1). According to the review [25], nanocracks approximately 20 nm in size may form at the Ni–PTFE interface due to the development of internal stresses during cooling. To improve interfacial contact, polymer particles are modified with surfactants, in particular the cationic surfactant CTAB, which promotes improved wettability and closer contact between the metallic matrix and the particles [119]. In study [116], the formation of a superhydrophobic Ni–PTFE–SiC coating with no visually detectable defects at the interface was achieved through optimization of the process parameters. Nanoindentation data indicate a possible local decrease in hardness near the interfacial region. This effect is attributed to the presence of microvoids; however, its magnitude is substantially reduced when particles smaller than 1 μm are used [116,119,120].

In cold spray, the interfacial boundary is formed predominantly through mechanical interlocking: the metallic matrix deforms plastically upon impact, while the polymer component is retained in pores, crevices, and interparticle spaces. The formation of chemical bonds is unlikely in this case due to the relatively low process temperature. Nevertheless, as demonstrated in study [97] for the Cu–polyamide system, sufficient cohesion can be achieved even without plastic deformation of the copper particles, which retained their spherical morphology; the bonding was provided by the polymeric binder and by mechanical interlocking at the metal–polymer boundary. Study [96] showed that in Cu–Zn/PTFE composite coatings produced by cold spray followed by friction treatment and annealing, the fluoropolymer component effectively fills structural defects and pores in the metallic matrix, creating an effective barrier against electrolyte penetration. The formation of needle-like oxide crystallites during annealing at 500 °C creates a developed surface relief that promotes additional mechanical interlocking of the polymer.

In thermal spray, the high processing temperatures (1500–3000 °C) can promote the formation of chemical bonds at the metal–polymer interface, particularly when heat-resistant polymers (PEEK, polyimides) are used. Study [89] demonstrated that flame-sprayed PEEK coatings on low-carbon steel achieved an adhesion strength of 16 ± 3 MPa, which is substantially higher than typical values for cold-sprayed metal–polymer coatings. This higher adhesion is attributed to the partial melting of the polymer and its interaction with the metal oxide layer during deposition. However, the thermal degradation of less thermally stable polymers (e.g., PTFE) during thermal spray limits the applicability of this method and may result in the formation of a weak, degraded interfacial layer [90].

In hybrid methods, the interfacial bonding is predominantly mechanical. During capillary impregnation of porous metallic coatings with polymer compositions, the polymer fills the pore network of the metallic matrix, forming a mechanical interlock upon solidification [98,99,100]. In UAM, plastic flow of the metal during ultrasonic welding envelops polymer inclusions, creating a strong mechanical bond [101,102]. In all cases of low-temperature hybrid processing, the formation of chemical bonds between the polymer and metal is unlikely without additional surface modification. The use of intermediate coupling layers (silanes, PDA, diazonium compounds) in hybrid methods represents an unexplored but promising direction for enhancing interfacial adhesion.

In summary, achieving reliable adhesion at the metal/polymer interface in MMPC coatings requires a deliberate, system-specific approach that integrates an understanding of the dominant adhesion mechanism with an appropriate surface treatment or functionalization strategy. The selection of an optimal strategy depends on the metal matrix material, the polymer filler type, the fabrication method, and the intended service conditions. For the most widely studied system, Ni–PTFE, surfactant modification improves particle incorporation but does not eliminate the fundamental adhesion problem; plasma treatment and chemical etching offer effective surface activation but face scalability challenges; and silane coupling and emerging grafting techniques provide the most promising route to durable chemical bonding but require further investigation in the context of MMPC coatings. The development of graded interfacial layers that provide a gradual transition from the metallic matrix to the polymer filler, combined with advances in low-temperature chemical grafting, represents the most promising direction for resolving the interfacial adhesion challenge in next-generation MMPC coatings.

## 5. Properties of MMPC Coatings

MMPCs have attracted considerable attention in materials science due to the possibility of flexibly controlling their performance characteristics by varying their composition and structure. The incorporation of dispersed fillers into a metallic matrix makes it possible to simultaneously modify the tribological, corrosion, mechanical, and functional properties of coatings, resulting in materials with a comprehensive set of advantages. A systematic analysis of the key properties of such composites, supported by experimental data from the recent scientific literature, is presented below.

### 5.1. Tribological Properties

One of the most pronounced effects of incorporating polymer fillers into metallic matrices is a substantial reduction in the coefficient of friction (COF). This is attributed to the inherent lubricating ability of polymer additives, as well as the formation of protective tribofilms and transfer layers on the contact surface. During friction, polymer particles deform and stretch, forming a lubricating layer; at the same time, the reduction in surface energy characteristic, for example, of PTFE decreases the adhesive component of friction, providing high antifriction performance of the composites [25,121,122].

A significant improvement in antifriction characteristics has been experimentally confirmed for a number of systems. In electroplated Ni–W/PTFE coatings, at an optimal PTFE concentration of 15 g/L, the COF decreases from 0.42 for the base Ni–W alloy to 0.24. In electrolessly deposited Ni–PTFE coatings containing 20–25 vol.% PTFE, the COF is 0.1–0.12, which is substantially lower than the values of 0.4–0.5 typical of pure nickel [123]. In the Cu–PTFE system (22 vol.% PTFE), the COF decreases from 0.55 to 0.13–0.25 [29].

According to the review [124], the incorporation of nanofillers into copper-, nickel-, and tungsten-based matrices makes it possible to substantially reduce the coefficient of friction and wear rate. The effect depends on the nature and concentration of the additive. So, for electrically conductive polymers in nickel-matrix composites, such as polyaniline and polypyrrole, friction reduction is achieved through a combination of lubricating action and stabilization of the interfacial boundary. At the same time, there is an optimal filler concentration range: exceeding the threshold value leads to particle agglomeration and accelerated pull-out, which offsets the beneficial effect. In a number of systems, such as Ni matrices with polymer additives, the COF was reduced by 40–60% compared with base metallic coatings, while wear was reduced by a factor of 2–3 when the deposition conditions and preliminary filler dispersion were properly selected.

**Figure 6:**
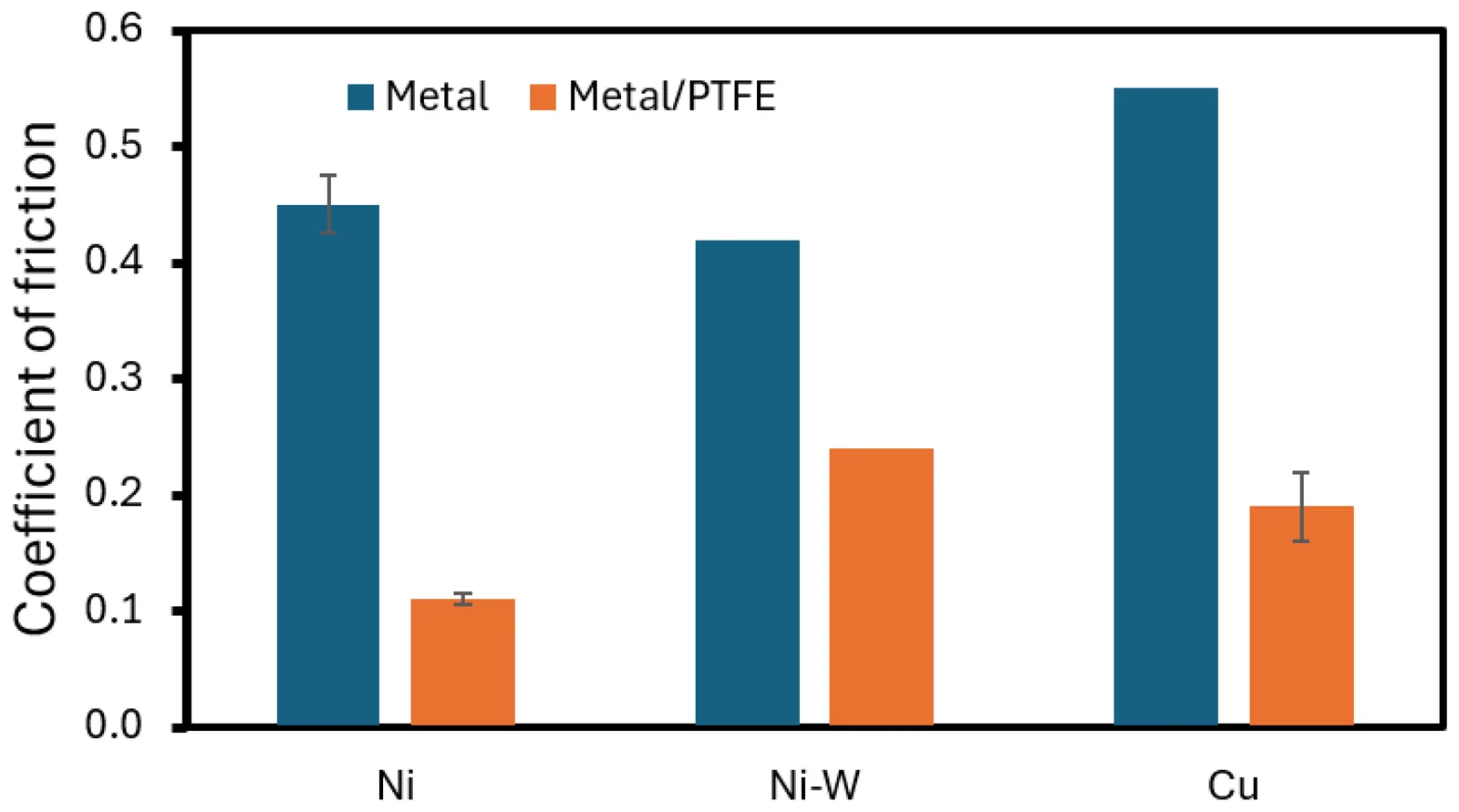
Effect of PTFE incorporation on the coefficient of friction.

An important parameter alongside the coefficient of friction is the wear resistance of the coatings (Figure 6).

For Ni–W/PTFE with a PTFE concentration of 15 g/L, the wear rate is 1.77 × 10^−7^ mm^3^/(N × m). Under oil-lubricated conditions, Cu–PTFE (22 vol.%) demonstrates a 57% reduction in mass loss compared with NiP–PTFE [29]. Filler particle size also has a substantial effect on wear resistance: Ni–PTFE nanocomposites containing PTFE particles 117 nm in size outperform microcomposites with 1 μm particles due to the combination of increased hardness and reduced surface energy [122].

Thus, the tribological characteristics of MMPC coatings are determined by a combination of factors, including the nature, concentration, and particle size of the filler, as well as the fabrication technology. Optimal antifriction and wear-resistant properties are achieved at a balanced additive concentration that promotes the formation of a stable tribofilm without excessive particle agglomeration, together with the use of technological approaches that ensure uniform filler distribution and improve interfacial interactions.

### 5.2. Corrosion Resistance

The corrosion resistance of MMPCs is determined by a combination of interrelated factors, including the barrier properties of the polymer phase, coating porosity, quality of the interfacial boundary, and electrochemical compatibility of the phases.

The “tortuous path effect” formed by polymeric and layered fillers (PTFE, etc.) plays an important role in protecting the substrate: such fillers substantially increase the diffusion paths of aggressive species toward the metallic substrate. The effectiveness of this approach is confirmed by quantitative measurements. For example, Cu–Zn/PTFE composite coatings on St3 steel, in which the fluoropolymer component fills structural defects and pores in the metallic matrix creating an effective barrier against electrolyte penetration, exhibit a corrosion current density of 8.5 × A/cm^2^ and an impedance modulus of |Z| = 5.3 × 10^4^ Ω × cm^2^, representing a 25-fold improvement compared with the reference Cu–Zn coating [96]. Optimization of the filler concentration is critically important, as it directly affects coating porosity and, consequently, its protective properties. Moderate incorporation of polymer particles (within approximately 25 vol.%) promotes the filling of microdefects and reduces pore density, thereby enhancing the barrier properties. Exceeding this threshold, as well as non-uniform filler distribution, promotes particle agglomeration, formation of microvoids, and through-porosity, which become sites of localized corrosion [127]. Practical confirmation is provided by salt spray testing of electrolessly deposited Ni–PTFE coatings containing 23–25 vol.% PTFE: at a thickness of 7.6 μm, these coatings withstand 100 h without signs of through-corrosion. A further increase in the PTFE fraction results in a sharp deterioration of the protective properties, highlighting the need for strict control of the coating composition [123].

The electrochemical compatibility of the phases also has a substantial effect on corrosion resistance. When interfacial adhesion is weak, combining components with different electrochemical potentials can lead to the formation of galvanic couples that accelerate matrix corrosion. This is particularly important when electrically conductive polymers such as polyaniline and polypyrrole are used. Under optimal conditions, with uniform particle distribution and moderate filler concentration, these polymers can act as corrosion inhibitors through passivation of the metallic surface: several studies have reported a 3–5-fold increase in polarization resistance and a shift of the corrosion potential toward more positive values. However, localized particle agglomeration and high coating porosity may promote galvanic corrosion, offsetting the beneficial effect [127].

The hydrophobicity of the filler surface is another factor that enhances corrosion resistance by reducing wetting and slowing electrolyte penetration toward the substrate. Fluorine-containing additives, as well as PTFE and epoxy systems, reduce coating permeability to chloride ions and water, as confirmed by electrochemical impedance spectroscopy and polarization measurements [26,128].

Thus, achieving high corrosion resistance requires more than simply selecting an appropriate filler; comprehensive control of the technological parameters during MMPC coating formation is necessary. Uniform particle distribution, minimization of porosity and through-defects, and optimization of interfacial interactions are of key importance. It is the combined effect of these characteristics that determines the durability of the coating’s protective action in aggressive environments.

### 5.3. Mechanical Properties

The mechanical properties of MMPC coatings are determined by the competition between two effects: the strengthening effect of dispersed particles and the weakening effect of soft polymer additives. The effect of the filler on hardness is non-monotonic and depends substantially on its nature.

**Figure 7:**
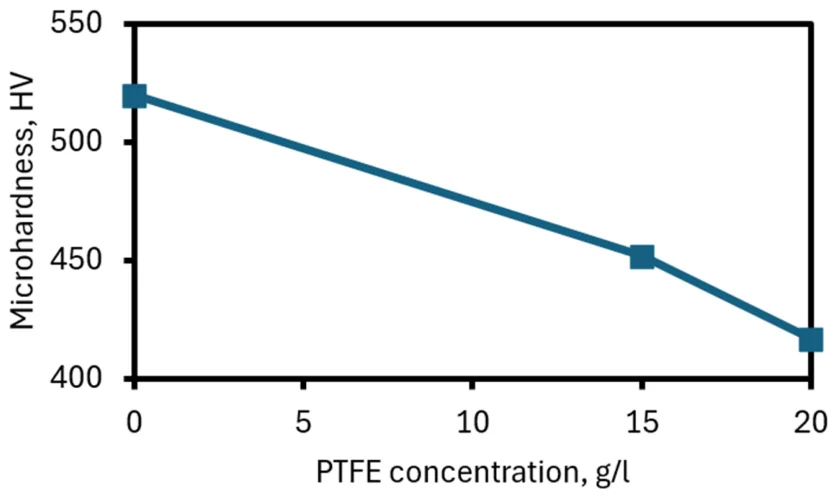
Effect of PTFE concentration in the deposition bath on the mechanical properties of Ni–W coatings.

In systems containing soft polymers such as PTFE, increasing the additive concentration leads to a decrease in hardness (Figure 7).

Thus, in Ni–W/PTFE coatings, the hardness decreases from 520 HV at 0 g/L PTFE to 452 HV at 15 g/L and 417 HV at 20 g/L due to the predominance of the softening effect of PTFE. The incorporation of nanoparticles into metallic matrices (Cu, Ni, W, and their alloys) provides strengthening through dispersion strengthening and the Orowan mechanism. The hardness of the coatings increases with filler concentration up to a certain limit, beyond which the properties begin to deteriorate due to increased porosity and the formation of structural defects. Systems containing polymer fillers (PTFE and epoxy resins) are characterized by a trade-off between hardness and ductility: the polymer reduces the absolute hardness but increases resistance to fatigue failure and crack propagation by damping local stresses [127].

The uniformity of filler distribution and the quality of interfacial interactions play an important role. When the bonding between the filler and matrix is weak, the particles become crack initiation sites and accelerate coating failure. Optimal mechanical properties are achieved through uniform filler distribution and the use of technological approaches that enhance interfacial bonding, such as particle surface modification, the use of surfactants, pulsed deposition, and ultrasonic treatment of the electrolyte [25,127].

The adhesion strength of coatings directly depends on the quality of the phase boundary. Typical pull-off strength values for industrial metal–polymer coatings are 3–12 MPa. At the same time, systems with a high particle content, such as Ni–PTFE, may develop nanocracks (~20 nm) due to internal stresses, which adversely affects coating durability [25,129].

The fatigue resistance of composites is determined by the ability of the material to inhibit crack propagation: dispersed particles can act as barriers, deflecting the crack path and slowing its growth. However, an excessive amount of polymer filler can reduce the fatigue life of the coating due to weakening of the metallic matrix [127]. It should be noted that data on the fatigue characteristics of MMPCs are limited in the scientific literature, highlighting the need for further research in this area.

Thus, the mechanical properties of MMPC coatings are determined by a complex balance of structural and technological parameters. The key factors include the nature and concentration of the filler, the uniformity of its distribution within the matrix, and the strength of the interfacial boundary. These parameters determine the balance between hardness, ductility, and fatigue resistance of the material.

### 5.4. Functional Properties

Modern composite coatings are increasingly being developed not only to improve traditional mechanical and corrosion characteristics but also to impart specialized functional properties to surfaces. The key functional characteristics of MMPCs and approaches to controlling them through the selection of the filler and deposition technology are considered below:

- Hydrophobicity and superhydrophobicity. The incorporation of hydrophobic components (PTFE, PDMS) makes it possible to substantially increase the water contact angle (WCA), in some cases reaching the superhydrophobic state. Smooth PTFE-containing coatings provide a WCA in the range of 108–114°. This effect can be significantly enhanced through microstructuring: for example, microstructured Ni–PTFE coatings achieve a WCA of 152°, while PDMS-based composites reach up to 165° [25,128,130]. Such properties provide a self-cleaning effect and additionally enhance corrosion resistance by minimizing surface contact with the aggressive environment.
- Anti-icing properties. Superhydrophobic coatings with a WCA > 150° and low surface energy effectively reduce ice adhesion to the surface. It has been experimentally demonstrated that such MMPCs reduce ice adhesion by 40–70% compared with smooth metallic surfaces [128,130], opening up prospects for their application under low-temperature climatic conditions.
- Electrical conductivity. The metallic matrix largely retains its electrical conductivity; however, the incorporation of non-conductive fillers (PTFE, epoxy resins) results in a gradual decrease in conductivity. For electrolessly deposited Ni–PTFE coatings containing 23–25 vol.% PTFE, the electrical resistivity is approximately 0.06 Ω × m at a thickness of 21 μm [123]. Upon reaching the percolation threshold (approximately 30–40 vol.%), the coating transitions to a dielectric state. At the same time, the use of conductive polymers (for example, polyaniline) can help maintain or even increase the electrical conductivity of the composite [122,127].
- Thermal conductivity. The thermal properties of MMPCs depend substantially on the type and fraction of the incorporated filler. The low thermal conductivity of PTFE (approximately 0.25 W/(m × K)) results in a decrease in the thermal conductivity of the composite proportional to the volume fraction of the polymer filler, making such materials promising for use as thermal barrier coatings.
- Biocompatibility. Biocompatible polymers, such as polycaprolactone, have been widely explored as scaffold materials, drug-delivery systems, and polymer-on-metal coatings on titanium and magnesium substrates for biomedical applications [51,52,53,124,127]. However, these systems are polymer-on-metal configurations that fall outside the strict MMPC definition: PCL has not yet been demonstrated as a dispersed filler within a continuous metallic matrix. The incorporation of PCL into MMPC coatings for medical implants remains a prospective but unexplored direction, where the load-bearing capacity of a metal matrix could potentially be combined with the biocompatibility and biodegradability of PCL [124,127].

Thus, the incorporation of polymeric and layered fillers into metallic matrices makes it possible to deliberately modify a wide range of properties of composite coatings. Functional characteristics, including hydrophobicity, anti-icing performance, and tunable electrical and thermal conductivity, substantially expand the potential applications of such coatings. At the same time, their tribological and corrosion properties are improved through reduced friction, increased wear resistance, and implementation of a barrier effect, provided that the composition is optimized and porosity is minimized. The mechanical properties exhibit a complex dependence on the nature and concentration of the filler: soft polymers may reduce hardness, whereas oxide particles provide strengthening.

Achieving an optimal balance between different properties requires careful selection of the composition, structure, and deposition technology of the composites. At the same time, a number of aspects, particularly fatigue resistance, remain insufficiently studied, highlighting the need for further research in this area.

The review of experimental data shows that PTFE-containing coatings consistently demonstrate the best tribological performance, with coefficient of friction reductions of 40–60% and wear rate reductions of 2–3× compared with pure metal coatings when the filler is properly dispersed and the concentration is optimized. For corrosion resistance, the key factors are uniform filler distribution, minimized porosity, and strong interfacial bonding rather than the filler type alone; conductive polymers (polyaniline, polypyrrole) can provide a 3–5-fold increase in polarization resistance under optimal conditions but may promote galvanic corrosion if agglomerated. Mechanical properties exhibit a non-monotonic dependence on filler content: soft polymers reduce hardness, while the strengthening effect is limited by weak interfacial bonding. Among functional properties, PTFE-based coatings achieve superhydrophobicity (WCA up to 152–165°) and reduce ice adhesion by 40–70%, making them the most versatile filler for multifunctional applications. Conductive polymer fillers can help maintain electrical conductivity, whereas insulating fillers cause a transition to a dielectric state at ~30–40 vol.%.

## 6. Effect of Composition and Fabrication Technology on the Properties of MMPC Coatings

The performance characteristics of metal–polymer composite coatings are determined by a combination of interrelated factors, including the nature of the metallic matrix and polymer filler, their quantitative ratio, and the specific processing conditions used for coating formation and subsequent treatment. Rational selection of each of these parameters makes it possible to deliberately control the physicomechanical, tribological, corrosion, and functional properties of the final material. A systematic analysis of the effects of the key factors on composite properties, supported by data from recent studies, is presented below:

- Matrix material—the choice of metallic matrix largely determines the baseline level of the coating’s functional characteristics and the available deposition methods. Nickel is the most widely used matrix in composite coatings due to well-established electroplating technologies and relatively high hardness, ranging from 200 to 600 HV [24,34,63,75,126]. A copper matrix is preferred when high thermal and electrical conductivity of the coating are the primary requirements [97]. Cobalt and its alloys offer high hardness and thermal stability, making them potentially suitable for high-temperature applications; however, cobalt-based MMPCs with polymer fillers remain largely unexplored. Titanium is used in the aerospace and medical industries, where high specific strength and biocompatibility are critically important [46]. Zinc and aluminum are traditionally used for corrosion protection; however, composite coatings based on these metals with polymer fillers remain relatively poorly studied [9,23,24,36,37,38,39,40,41].
- Polymer filler—the type of polymer filler determines the specific functional properties of the composite. Polytetrafluoroethylene (PTFE) serves as the primary antifriction component, providing a low coefficient of friction in the range of 0.05–0.1 and retaining its performance at temperatures up to 250–300 °C [46,112]. Epoxy resins are used to improve adhesion and increase corrosion resistance in metal matrix composite coatings, particularly in zinc- and aluminum-based systems [18,49,50,53]. Polyimides (PAI) are valued for their high thermal stability (up to 300 °C), which extends the temperature range of coating applications [18,86,97]. Conductive polymers, such as polyaniline and polypyrrole, make it possible to impart the required electrical properties to composites [54,55]. In biomedical applications, polycaprolactone has been extensively studied as a scaffold material and as a polymer-on-metal coating for implants [51,52,53]. However, these studies address PCL in the context of tissue engineering and drug-delivery systems rather than genuine MMPC coatings. The use of PCL as a dispersed filler incorporated into a continuous metal matrix has not been demonstrated and remains a prospective direction for future MMPC research.
- Filler volume fraction—the concentration of the dispersed phase in the composite is one of the key parameters determining the balance of its performance characteristics. For PTFE, a content of 5–25 vol.% is generally considered optimal. In particular, for the Ni–W/PTFE system, the best tribological characteristics are achieved at a concentration of 9.4 wt.% (corresponding to 15 g/L in the electrolyte) [25,75]. Exceeding the optimal filler content leads to undesirable effects, including particle agglomeration and increased coating porosity, and consequently deterioration of the mechanical, tribological, and corrosion properties. Therefore, strict control of the filler volume fraction is necessary to ensure the stability and reproducibility of composite coating characteristics.
- Pretreatment and deposition conditions—process parameters substantially affect the structure and properties of the resulting coating. In study [82], a two-stage approach to the fabrication of Ni–P–PTFE composite coatings was employed: the first stage (20 min) involved static immersion of the sample in the plating bath to ensure effective adsorption of PTFE particles onto the substrate surface, while the second stage (20 min) was carried out under intensive agitation (200 rpm at 88 °C), resulting in a polymer mass fraction of 25.96% with uniform particle distribution throughout the coating thickness. Pulse electroplating at a low duty cycle (10–30%) and a pulse frequency of 10–100 Hz makes it possible to approximately double the degree of particle incorporation into the matrix compared with direct-current deposition [73,75,76]. Ultrasonic agitation promotes more effective particle dispersion in the electrolyte, reducing the probability of agglomeration and ensuring uniform filler distribution within the coating [8,101,102]. An alternative method for depositing polymer-containing coatings without the risk of thermal degradation of the polymer is cold spray, which preserves the initial properties of thermally sensitive components [7,91,93,96,113].
- Post-treatment—heat treatment of composite coatings is an effective tool for deliberately controlling their performance characteristics. Variation in the temperature regime can substantially modify the structural state of the material and, consequently, its mechanical properties. In particular, heating promotes phase transformations, including crystallization of amorphous phases, and redistribution of internal stresses, which directly affects the hardness and wear resistance of the coating. Optimization of heat-treatment parameters is an important stage in coating design, ensuring the required balance of strength and functional characteristics [10,11,20,112].

The analysis confirms that the performance of MMPC coatings is determined by the combined effect of matrix–filler pair, filler volume fraction, particle size, deposition method, and post-treatment. For PTFE-filled systems, an optimal content of 5–25 vol.% is consistently reported; for the Ni–W/PTFE system specifically, the best tribological characteristics are achieved at ~9.4 wt.% (15 g/L in the electrolyte). Heat treatment is an effective tool for tailoring mechanical properties through phase transformations and redistribution of internal stresses, but its parameters must be carefully optimized to avoid degradation of the polymer phase. The overall conclusion is that no single parameter dominates; instead, a balanced optimization of composition, processing conditions, and post-treatment is required to achieve the target combination of tribological, corrosion, and mechanical properties.

## 7. Main Application Areas of MMPC Coatings

The multifunctionality of MMPCs makes them attractive for a wide range of high-tech industrial sectors. The combination of high wear resistance, corrosion resistance, tunable tribological characteristics, and specialized functional properties enables the use of such coatings under increased mechanical, thermal, and chemical loads. Thus, in mechanical engineering, MMPC coatings are successfully used to produce antifriction components with a reduced coefficient of friction and increased wear resistance. Protective coatings for components operating in aggressive environments are of particular importance, as are repair coatings for restoring expensive equipment. Corrosion-resistant coatings provide reliable protection against exposure to operating environments [8,9,18,27,63,108,122]. The aerospace industry actively uses heat-resistant coatings for engine components and cooling systems. Lightweight composite materials make it possible to substantially reduce structural weight while maintaining strength characteristics. Anti-icing coatings protect aircraft surfaces, while wear-resistant materials are used for landing gear components and control mechanisms [4,22,31,68]. In the oil and gas industry, a priority area is the development of corrosion-resistant coatings for protection against petroleum products and saline solutions. Abrasion-resistant coatings provide protection during fluid transport, sealing materials are used for sealing components, and antifriction coatings are applied to pump equipment [12,19,21,96,100,109].

The medical industry widely uses biocompatible coatings for implants and prostheses, as well as antibacterial materials for medical instruments. Hydrophobic coatings protect surfaces from biological fluids, while electrically conductive materials are used in modern medical devices [51,52,53,115,125,128]. It should be emphasized that the cited biomedical literature on polycaprolactone, including scaffold fabrication, drug-delivery systems, and PCL coatings applied onto metallic substrates [51,52,53], describes polymer-on-metal systems (outside the strict MMPC definition) and tissue-engineering constructs rather than genuine metal-matrix/PCL composite coatings. To date, no true MMPC coating with PCL as a dispersed filler in a continuous metallic matrix has been reported. The development of such coatings, combining the mechanical strength of a metal matrix with the biocompatibility and biodegradability of PCL, represents a promising but unrealized direction in the field of medical MMPCs. The electronics industry is actively implementing protective coatings for microchips and electronic components. Conductive composites are used in contacts and interconnects, heat-dissipating materials provide effective cooling of electronic components, while insulating coatings offer tunable dielectric properties [23,101,103]. In the energy sector, fire-resistant coatings are used for components of energy-generation systems, while thermal barrier materials provide protection against high temperatures. Anti-corrosion coatings ensure reliable protection of equipment operating in aggressive environments, and electrical insulating materials are used in power equipment [11,59,87,109,114].

Current technological development trends include the implementation of digital manufacturing using additive technologies, the creation of “smart” equipment with integrated functional coatings, and the development of environmentally friendly technologies based on biodegradable polymers. Particular attention is being paid to personalized manufacturing tailored to specific customer requirements [51,52,68,94,101,102,106].

Thus, MMPC coatings have broad applications across various industrial sectors. Their multifunctionality is determined by the possibility of flexibly controlling their properties by varying the matrix and filler composition and optimizing the deposition parameters. Expansion of the application range of MMPCs is associated with the development of new compositions and deposition technologies that make it possible to combine within a single material a set of performance characteristics previously achievable only through specialized solutions. Further research should focus on improving coating durability under extreme conditions, optimizing their composition for specific applications, and developing cost-effective methods for industrial-scale production, which will help realize the full potential of MMPCs in high-tech industries.

## 8. Current Research Trends in MMPC Coatings

The development of materials science in the field of composite coatings is characterized by a shift in focus from achieving individual performance characteristics toward the development of multifunctional and “smart” materials capable of adapting to service conditions, restoring their properties, and minimizing their environmental impact. Current research trends include the transition to nanoscale fillers, the development of multiphase and self-healing systems, the fabrication of coatings with tunable properties, and the implementation of environmentally friendly technologies. The key research directions in the field of MMPC coatings are discussed below:

- Nanoscale polymer fillers—one of the most significant trends is the transition from the use of microparticles to nanoscale fillers. The use of PTFE nanoparticles, for example, with a size of 117 nm, makes it possible to achieve higher hardness and wear resistance at substantially lower filler concentrations compared with microdispersed analogues [25,131,132]. This approach not only improves the mechanical characteristics of composites but also enhances their barrier properties due to denser particle packing and a reduction in structural defects. In addition, nanoscale fillers promote a more uniform distribution of stresses within the matrix, which has a positive effect on coating durability [133,134].
- Multiphase coatings—combining several types of fillers within a single matrix provides opportunities for achieving a synergistic effect, in which the overall properties of the composite exceed the sum of the effects of the individual components [135,136]. Examples of such systems include Ni–P–Al_2_O_3_–PTFE coatings, in which PTFE provides self-lubrication while Al_2_O_3_ contributes to dispersion strengthening and wear resistance, demonstrating a synergistic improvement in both tribological and mechanical characteristics [75]. The multiphase approach makes it possible to address several service requirements simultaneously: achieving a low coefficient of friction, increasing wear resistance, improving barrier properties, and stabilizing the coating structure under thermal and mechanical loads [56].
- Self-healing coatings—the development of this type of coating is aimed at increasing the durability and reliability of composite materials through their ability to autonomously repair damage. Existing solutions can be divided into extrinsic and intrinsic healing systems. Extrinsic healing systems are implemented by incorporating microcapsules containing healing agents directly into the metallic matrix. When mechanical damage occurs, the capsules rupture, releasing the encapsulated substance and subsequently repairing the defect. Intrinsic healing systems operate through dynamic chemical bonds that enable the material to restore its structure autonomously. Such systems can provide repeated healing without loss of their initial properties or external intervention. Of particular interest is the technology of phase-separated metal–polymer systems, which demonstrate high healing efficiency. These innovative solutions combine a metallic framework with polymer components capable of self-organization upon damage. Studies have shown that healing efficiency in some systems can reach substantial values, opening up broad prospects for the application of these materials in various industrial sectors. A notable example is the development of self-healing polymer coatings based on polycaprolactone, where phase-separated PCL/epoxy blends demonstrate healing efficiencies of up to 90% upon thermal activation. However, all such systems reported to date are based exclusively on polymer matrices (e.g., epoxy) or polymer-on-metal coating architectures; none involve a continuous metallic matrix with dispersed PCL filler. Consequently, the implementation of self-healing functionality in genuine MMPC structures remains a prospective direction that has not yet been realized experimentally [137,138,139,140,141].
- Smart coatings with tunable properties—coatings capable of responding to changes in external conditions, including pH, temperature, electrochemical potential, and other parameters. Such solutions include omniphobic coatings resistant to various types of contamination; fluorescent damage markers that enable visualization of defects at an early stage; and coatings with tunable wettability that adapt to service conditions. Such systems expand the functional capabilities of composite coatings, transforming them from passive protective layers into active elements capable of monitoring their condition and optimizing their performance characteristics in real time [142,143,144,145].
- Environmentally friendly technologies—in recent years, substantially increased attention has been devoted to the environmental aspects of manufacturing processes. In the field of composite coating deposition, this is reflected in the transition from toxic technologies, such as those involving Cr^6+^, to safer alternatives, including Ni–W and Ni–Co coatings and phosphorus-free electrolytes. In parallel, “green” coating fabrication methods are being developed, including the use of aqueous systems instead of organic solvents, ultrasonic electroplating in supercritical CO_2_, and cold spray, which eliminates the generation of toxic waste. These approaches not only reduce the environmental impact but can also improve coating quality through more precise control of structure and composition [34,146,147,148,149].

Thus, current research on MMPC coatings is focused on the development of a new generation of materials with expanded functionality and improved service reliability. The transition to nanoscale fillers and multiphase systems makes it possible to achieve synergistic effects and substantially improve the mechanical, tribological, and barrier characteristics of coatings. The development of self-healing and “smart” technologies opens up new opportunities for autonomous monitoring of material condition and adaptation to changing service conditions. The implementation of environmentally friendly manufacturing methods is also an important aspect, consistent with current sustainability requirements. Further research should focus on scaling up promising laboratory-scale solutions, optimizing compositions and processing parameters for industrial applications, and developing comprehensive criteria for evaluating the durability and functionality of “smart” composite coatings. This will not only expand their range of applications but also improve the economic and environmental efficiency of MMPCs in high-tech industrial sectors.

## 9. Unresolved Problems and Development Prospects of MMPC Coatings

Despite significant progress in the development and application of metal–polymer composite materials, a number of fundamental and technological problems remain unresolved, limiting the scale-up of promising laboratory-scale solutions and their implementation in industrial practice. The key challenges are associated with ensuring reliable adhesion at the metal–polymer interface, achieving the required thermal stability of composites, scaling up coating deposition technologies, and the lack of standardized testing methods:

- Adhesion strength at the metal/polymer interface—one of the most critical problems in MMPC technology is ensuring strong bonding between the polymer filler and the metallic matrix. This problem is particularly pronounced when nonpolar polymers such as PTFE are used, as they are characterized by poor wettability of metallic surfaces, which substantially limits the adhesion strength of the composite. Partial solutions can be achieved through the use of surfactants, ultrasonic treatment, and silane modifiers; however, the reproducibility of these approaches remains insufficient. Chemical grafting of polymers to the metallic substrate appears to be a promising approach, for example, through anchoring using diazonium compounds (diazonium-induced anchoring). Another promising direction is the formation of graded interfacial layers that provide a gradual change in composition and properties from the metallic matrix to the polymer filler, thereby reducing internal stresses and increasing coating durability [1,39,83,116,117,118,119,120,127].
- Thermal stability of composite coatings—the limited thermal stability of certain polymer components substantially narrows the temperature range of MMPC applications. For example, PTFE undergoes thermal degradation at temperatures above 250–300 °C, making it unsuitable for high-temperature applications. Heat-resistant polymers such as polyimides, as well as hybrid fillers combining polymeric and inorganic components, are being considered as alternatives. However, it should be noted that in these systems the primary load-bearing phase is the metallic matrix rather than the polymer component, which fundamentally distinguishes such materials from conventional polymer-containing composites. The development of polymer and hybrid systems capable of retaining their functional properties at high temperatures remains one of the key challenges in materials science in this field [31,59,86,112,115,134,135].
- Scaling up coating deposition technologies—transferring laboratory-scale results to industrial production is associated with a number of technological difficulties. The main problems include instability of suspensions containing nanofillers, difficulties in ensuring uniform coating thickness on surfaces with complex geometries, and the high cost of nanoscale fillers, which limits the economic feasibility of their large-scale use. Overcoming these barriers requires the development and implementation of automated process control systems capable of precisely controlling key parameters, including particle concentration in the electrolyte, current density, temperature, and hydrodynamic conditions. Such systems will improve the reproducibility of coating characteristics and reduce the fraction of defective products in serial production [8,62,70,73,76,122,124].
- Standardization of testing methods—the lack of unified standards for evaluating the performance characteristics of MMPCs substantially complicates the comparison of results obtained at different research centers and slows the implementation of new materials in practice. At present, there are no standardized methods for determining tribological properties, accurately measuring filler volume fraction, or evaluating the corrosion resistance and adhesion strength of composite coatings. This leads to significant variation in the reported data and reduces the reliability of comparative analyses of different compositions and technologies. The development of dedicated standards within international organizations such as ISO and ASTM is necessary to ensure comparability of results, objective evaluation of the effectiveness of new solutions, and accelerated certification for industrial applications [9,12,58,121,122,127,129].

The review identifies three critical unresolved challenges that hinder the practical implementation of MMPC coatings. Weak adhesion at the metal–polymer interface remains the most fundamental problem, particularly in low-temperature deposition methods where bonding is limited to van der Waals interactions; while coupling agents and surfactants offer partial solutions, a universally effective strategy has not been established. The limited thermal stability of polymer fillers (e.g., PTFE degrades above 250–300 °C) restricts the operating temperature range, and although heat-resistant alternatives (polyimides, PEEK) exist, they do not fully resolve the problem for high-temperature applications. The lack of standardized testing methods for tribological properties, filler volume fraction measurement, and adhesion evaluation significantly complicates the comparison of results across studies and slows the certification of new materials. Additionally, scaling up from laboratory to industrial production faces technological barriers, including suspension instability with nanofillers and the high cost of nanoscale materials. Addressing these challenges will require coordinated efforts in interface engineering, development of new polymer systems, and the establishment of international testing standards.

## 10. Consolidated Results and Insights

To provide a consolidated overview of the key findings systematized in this review, Table 3 summarizes the principal aspects of MMPC coatings discussed across all sections, including classification, fabrication methods, structure–property relationships, functional properties, and unresolved challenges. This table serves as the basis for the overall summary and conclusions presented in this section.

As shown in Table 3, the field of MMPC coatings has matured significantly in terms of fabrication technology and fundamental understanding of structure–property relationships. However, the transition from laboratory-scale research to industrial implementation is constrained by the interfacial adhesion problem, thermal stability limitations, and the absence of standardized characterization methods. The most promising directions for future research include the development of graded interfacial layers with chemical grafting, nanostructured and multicomponent fillers, self-healing systems, and environmentally friendly fabrication processes.

## 11. Conclusions

Metal matrix polymer composites (MMPCs) represent a rapidly developing area of materials science that combines the advantages of metallic matrices (mechanical strength, electrical conductivity, and corrosion resistance) and polymer fillers (low coefficient of friction, chemical inertness, and functional adaptability). The systematic analysis presented in this review demonstrated that the choice of metallic matrix and polymer filler type critically determines the performance characteristics of the composite and the available fabrication methods.

Among the coating fabrication technologies considered, electroplating is the most widely used, providing precise control over coating thickness and composition at relatively low temperatures. Electroless plating expands the possibilities for coating components with complex geometries and non-metallic substrates. Thermal spray offers high productivity but is limited by the thermal instability of most polymers. Cold spray preserves the integrity of the polymer phase but requires substantial capital investment. Hybrid methods are particularly promising, as they can compensate for the limitations of individual approaches and enable the fabrication of coatings with tailored architectures.

It was established that filler particle size, concentration, and distribution within the matrix have a determining effect on the tribological, corrosion, and mechanical properties of the coatings. The optimal parameters vary depending on the fabrication method: nanoscale particles (50–500 nm) are preferred for electroplating, the submicron range (0.2–0.5 μm) for electroless plating, and particles 5–10 μm in size for cold spray. Exceeding the optimal filler concentration leads to agglomeration, increased porosity, and deterioration of the performance characteristics.

The multifunctionality of MMPCs makes them attractive for applications in mechanical engineering, aerospace, oil and gas, medical, electronics, and energy industries. Current research trends are focused on the transition to nanoscale fillers, the development of multiphase and self-healing systems, the development of “smart” coatings with tunable properties, and the implementation of environmentally friendly manufacturing technologies.

Despite significant progress, a number of fundamental and technological problems remain unresolved, including ensuring reliable adhesion at the metal/polymer interface, expanding the operating temperature range, scaling up coating deposition technologies, and the lack of standardized testing methods. Further development of the field requires a comprehensive approach combining methods for chemical modification of the interfacial boundary, the development of heat-resistant hybrid fillers, process automation, and standardization of methods for evaluating durability. Progress in these areas will promote the transition from laboratory prototypes to reliable and economically efficient industrial solutions.

## Figures and Tables

**Table 1:** Comparative characteristics of the methods considered for MMPC fabrication.

Method	Advantages	Limitations	Typical Matrices/Fillers	Processing Temp., Typical Thickness
Electrodeposition	Low processing temperature, control of thickness and composition, high coating–substrate adhesion, scalability	Agglomeration at high concentrations, risk of polymer degradation in the electrolyte, dimensional limitations of the bath	Ni, Cu, Co, Zn PTFE	40–60 °C 10–60 μm
Electroless Plating	Uniformity on complex-shaped components, no external current required, deposition onto non-metallic substrates	Low deposition rate, thickness ≤ 50 μm, need for surfactants and stabilizers	Ni-P, Cu PTFE	~85–90 °C ≤50 μm (<5 μm achievable)
Thermal Spraying	High productivity, ability to produce thick coatings, suitability for large-sized components	Thermal degradation of polymers with melting points below 300 °C, limited selection of heat-resistant polymers	Ni, Co, alloys PEEK, polyimides	polymer melting point > 300–350 °C; filler 10–50 μm, microcapsules 30–70 μm, total coating thickness up to several mm
Cold Spray	Preservation of the polymer phase, absence of thermal oxidation, deposition onto thermally sensitive substrates	High equipment cost, fragmentation of brittle polymers upon impact	Cu, Cu–Zn, Al PTFE, PA6, PEEK	up to ~327 °C up to ~1 mm
Hybrid methods	Synergistic effects, compensation for the limitations of individual methods, no aggressive surface pretreatment	Multistage processing, complexity of process synchronization, difficulty in scaling up	Cu, Al, Ni, bronze, stainless steel PTFE, PA6, PVDF, microcapsules (PU), epoxy resins	up to ~300 °C up to several mm
DED	Potential for functionally graded coatings	Polymer fillers not yet implemented, experimental stage	Ti, alloys —	>1000 °C not yet reported for MMPCs

**Table 2:** Summary of adhesion mechanisms at the metal/polymer interface.

Mechanism	Bond Energy	Typical Methods
Mechanical interlocking	No chemical bonds; depends on surface morphology	Cold spray, thermal spray, UAM [95,102,103,107,108,121,122,123,124,125]
Adsorption bonding	0.05–0.5 kJ/mol (van der Waals)	Electroplating, electroless plating, ultrasonic impregnation [42,80,81,84,86,87,113]
Hydrogen bonding	10–40 kJ/mol	All methods (requires polar groups) [126,127,128,129,130]
Chemical (covalent) bonding	200–800 kJ/mol	Thermal spray; low-temperature methods with coupling agents [126,127,128,130]

**Table 3:** Comprehensive summary of key findings in MMPC coatings research.

Aspect	Key Findings	Key References
Metallic matrices	Ni is the predominant matrix (hardness 200–600 HV, broad compatibility with fluoropolymers); Cu for conductivity/wear; Co for high-load tribology; Al for lightweight composites; Ti and Zn are promising but underexplored; Cr and Fe are practically absent	[24,25,26,27,28,29,30,31,32,33,34,35,36,37,38,39,40,41,42]
Polymer fillers	PTFE is the most widely used (antifriction); epoxy resins for adhesion/corrosion; PANI/PPy for conductivity/protection; PCL for biomedical (prospective, not yet realized as true MMPC filler)	[43,44,45,46,47,48,49,50,51,52,53,54,55,56,57,58,59,60]
Filler morphology	Particles (0.05–50 μm) dominate, with sub-micron sizes offering optimal hardness–friction balance; fibers (1–10 μm diameter) for reinforcement in powder metallurgy/thermal spray; microcapsules for self-healing (up to 81.9% COF reduction)	[26,61,62,63,64,65,66,67,68,69]
Fabrication methods	Electrodeposition: predominant (40–60 °C, 10–60 μm, scalable); electroless plating: complex shapes, non-metallic substrates, max 50 μm; thermal spray: thick coatings, heat-resistant polymers only (PEEK, polyimides); cold spray: preserves polymer integrity, high cost; hybrid methods: most promising, compensate individual limitations	[10,70,71,72,73,74,75,76,77,78,79,80,81,82,83,84,85,86,87,88,89,90,91,92,93,94,95,96,97,98,99,100,101,102]
Structure formation	Optimal particle size: sub-micron (electroplating), 0.2–0.5 μm (electroless), 10–50 μm (thermal spray); concentration control is critical—agglomeration above threshold (e.g., >15–20 g/L PTFE in electrolyte); pulse deposition and surfactants improve uniformity	[25,73,75,76,82,104]
Interfacial adhesion	Four mechanisms: mechanical interlocking, adsorption (van der Waals, <0.5 kJ/mol), hydrogen bonding, chemical bonding (200–800 kJ/mol); weak adhesion remains a key challenge, especially for PTFE; silane coupling and chemical grafting are the most promising strategies	[38,76,104,113,114,115,116,117,118,119,120,121]
Tribological properties	PTFE-filled coatings reduce COF by 40–60% and wear rate by 2–3×; optimal PTFE content 5–25 vol.% (for Ni–W/PTFE: ~9.4 wt.%, i.e., 15 g/L in electrolyte, COF = 0.24); heat treatment can further improve wear resistance	[26,75,122,123]
Corrosion resistance	Key factors: uniform filler distribution, minimized porosity, strong interfacial bonding; conductive polymers (PANI, PPy) provide 3–5× increase in polarization resistance; PTFE fills defects and blocks electrolyte penetration (|Z| up to 5.3 × 10^4^ Ω × cm^2^)	[9,54,96,125]
Functional properties	Superhydrophobicity (WCA up to 152–165°); anti-icing (40–70% ice adhesion reduction); self-healing via microcapsules (COF reduction up to 81.9%); biocompatibility (PCL-based, prospective)	[51,52,53,67,128,140]
Applications	Mechanical engineering (antifriction coatings, plain bearings); aerospace (wear/corrosion protection); oil & gas (pipeline protection); electronics (conductive coatings, EMI shielding); biomedical (implant coatings, prospective)	[4,7,8,9,22,124]
Unresolved challenges	Weak metal/polymer adhesion (no universal solution); limited thermal stability (PTFE degrades > 250–300 °C); scaling difficulties (suspension instability, cost of nanofillers); lack of standardized testing methods for tribology, coating–substrate adhesion, and filler volume fraction	[8,25,86,122,124,134,135]

## Data Availability

No new data were created or analyzed in this study. Data sharing is not applicable to this article.
